# DAP12 interacts with RER1 and is retained in the secretory pathway before assembly with TREM2

**DOI:** 10.1007/s00018-024-05298-w

**Published:** 2024-07-15

**Authors:** Yanxia Liu, Sandra Theil, Melanie Ibach, Jochen Walter

**Affiliations:** Department of Neurology, University of Bonn, Bonn, 53127 Germany

**Keywords:** Neuroinflammation, Microglia, Neurodegeneration, Alzheimer disease, Nasu-Hakola disease, Phosphorylation

## Abstract

DNAX-activating protein of 12 kDa (DAP12) is a transmembrane adapter protein expressed in lymphoid and myeloid lineage cells. It interacts with several immunoreceptors forming functional complexes that trigger intracellular signaling pathways. One of the DAP12 associated receptors is the triggering receptor expressed on myeloid cells 2 (TREM2). Mutations in both DAP12 and TREM2 have been linked to neurodegenerative diseases. However, mechanisms involved in the regulation of subcellular trafficking and turnover of these proteins are not well understood. Here, we demonstrate that proteasomal degradation of DAP12 is increased in the absence of TREM2. Interestingly, unassembled DAP12 is also retained in early secretory compartments, including the endoplasmic reticulum (ER) and the ER-Golgi intermediate compartment (ERGIC), thereby preventing its transport to the plasma membrane. We also show that unassembled DAP12 interacts with the retention in ER sorting receptor 1 (RER1). The deletion of endogenous RER1 decreases expression of functional TREM2-DAP12 complexes and membrane proximal signaling, and resulted in almost complete inhibition of phagocytic activity in THP-1 differentiated macrophage-like cells. These results indicate that RER1 acts as an important regulator of DAP12 containing immunoreceptor complexes and immune cell function.

**Graphical Abstract:**

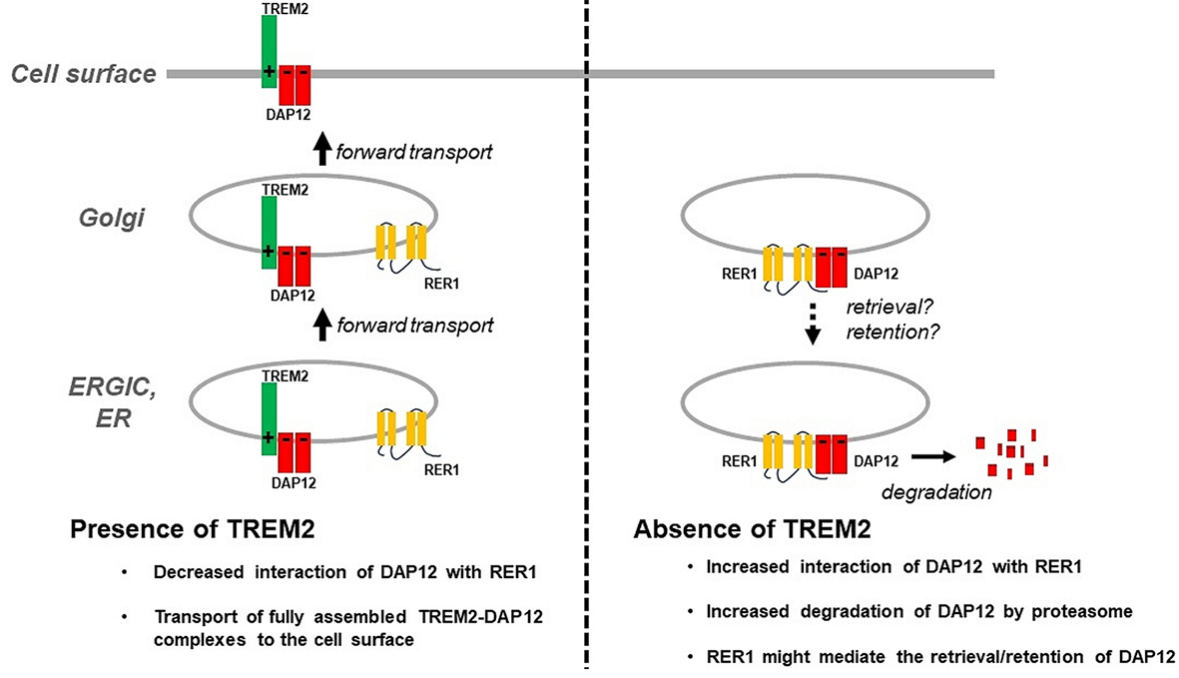

**Supplementary Information:**

The online version contains supplementary material available at 10.1007/s00018-024-05298-w.

## Introduction

DAP12, also known as tyrosine kinase-binding protein (TYROBP), is a transmembrane adapter protein for several immunoreceptors, and expressed in lymphoid and myeloid lineage cells, including natural killer cells, monocytes/macrophages, dendritic cells, osteoclasts, and microglia [1–4]. DAP12 has a very short ectodomain containing two cysteine residues that covalently link two monomers into a homodimer by disulfide bonds. The transmembrane domain (TMD) of DAP12 contains a charged aspartic acid residue at position 50 (D50), which interacts with the corresponding basic residue (lysine or arginine) of its associated receptors [5–7]. DAP12 containing immunoreceptor complexes are transported in the secretory pathway to the plasma membrane, and can be internalized into endocytic vesicles [8–11]. The cytoplasmic domain of DAP12 contains a classical immunoreceptor tyrosine based activating motif (ITAM) that mediates signal transduction upon ligand interaction [12, 13].

One of the immunoreceptors that assembles with DAP12 is TREM2, a member of the immunoglobulin-lectin like superfamily of receptors with a type I membrane protein topology [14–18]. The N-terminal ectodomain includes a ligand-binding Ig-like v-set domain. The transmembrane domain contains a lysine residue at position 186 (K186) for electrostatic interaction with DAP12, while the short C-terminal cytoplasmic domain has no known function for signal transmission. The binding of ligands to TREM2 can induce the phosphorylation of DAP12 at characteristic tyrosine residues in the ITAM, thereby triggering the recruitment and phosphorylation of the spleen tyrosine kinase (SYK). SYK regulates several signaling pathways involved in cell survival and proliferation, phagocytosis, chemotaxis, cytoskeletal remodeling, calcium mobilization and cytokine production [19–22]. Loss-of-function mutations of TREM2 or DAP12 cause Nasu-Hakola disease (NHD), also named polycystic lipomembranous osteodysplasia with sclerosing leukoencephalopathy (PLOSL), a very rare inherited severe neurodegenerative disease that is associated with bone fractures, frontal lobe syndrome, progressive presenile dementia and early death in the fourth or fifth decade of life [23–26]. Interestingly, one of the NHD pathogenic mutations is a substitution of a lysine residue (K) by an asparagine residue (N) at position 186 in the TMD of TREM2 that impairs the interaction with DAP12, indicating the importance of proper association of both proteins in immune cell function [27]. However, the regulation and mechanisms underlying complex assembly of DAP12 with its associated receptors is far from being completely understood. In particular, the subcellular trafficking and dynamics of receptor complex assembly remain to be characterized. The prevailing view is that the assembly of DAP12-receptor complexes in the ER involves at least three steps: (1) co-translational membrane insertion of DAP12 in the ER, (2) formation of DAP12 homodimers and (3) assembly with the respective immunoreceptor [28]. In the absence of an appropriate immunoreceptor, DAP12 tends to form trimers or tetramers with the negatively charged aspartate residues possibly coordinating cations. The DAP12 homotrimers and homotetramers might represent intermediate assembly products that compete with formation of the DAP12-receptor heterotrimer in the ER [28]. Previous studies suggested that DAP12 promotes expression of TREM2 or the two-domain-stimulatory killer Ig-like receptor (KIR2DS) at the cell surface [11, 29], although DAP12 appears not necessary for surface localization of TREM2 and KIR2DS [30, 31]. RER1 acts as a receptor recognizing specific features in the transmembrane domains of unassembled or misfolded proteins thereby ensuring their retention in or retrieval to the ER before successful assembly with other complex components [32–35].

Here, we analyzed molecular mechanisms that regulate the assembly and subcellular transport of the TREM2-DAP12 complex. Our data demonstrate retention and/or retrieval of DAP12 in the ER and ERGIC in the absence of TREM2. The aspartic acid residue in the TMD is critical for the retention of unassembled DAP12 and its association with RER1. Genetic deletion of RER1 revealed that this protein controls cellular levels of functional TREM2-DAP12 complexes and phagocytic activity of macrophage-like THP-1 cells. Thus, the combined results provide insight into the molecular mechanisms underlying the assembly and transport of DAP12 containing immunoreceptor complexes, and identified RER1 as an important regulator of immune cell function.

## Materials and methods

**Table:** 

Reagent/resource	Reference or source	Identifier or catalog number
**Chemicals/Reagents**		
Phorbol 12-myristate 13-acetate (PMA)	Sigma-Aldrich	Cat# P8139
Cycloheximide (CHX)	Sigma-Aldrich	Cat# C4859-1ML
MG132	Sigma-Aldrich	Cat# C2211-5MG
Lactacystin	Sigma-Aldrich	Cat# L6785-.2MG
Chloroquine	Sigma-Aldrich	Cat# C6628-25G
β-Mercaptoethanol	Sigma-Aldrich	Cat# M7522
N-dodecyl-β-d-Maltoside	Sigma-Aldrich	Cat# D4641
Protease inhibitor cocktail	Sigma-Aldrich	Cat# 04693116001
Phosphatase inhibitor cocktail	Sigma-Aldrich	Cat# 4906837,001
Poly-L-lysine (PLL)	Sigma-Aldrich	Cat# P6282
Cytochalasin D	Sigma-Aldrich	Cat# C8273
Lipofectamine 2000	Thermo Fisher Scientific	Cat# 11668-019
Hygromycin B	InvivoGen	Cat# ant-hg-5
Fetal Bovine Serum	PAN-Biotech	Cat# P30-3306
Penicillin/streptomycin (P/S)	Gibco	Cat# 15140-122
Cas9 enzyme	Integrated DNA Technologies (IDT)	Cat# 1081061
Lipofectamine RNAiMAX	Invitrogen	Cat# 13778-150
Paraformaldehyde (PFA)	Sigma-Aldrich	Cat# 16005-1KG-R
Triton X-100	Carl Roth	Cat# 3051.2
Tween® 20	Sigma-Aldrich	Cat# P1379
Immu-Mount™	Fisher Scientific	Cat# 9990402
Ibidi mounting medium	Ibidi	Cat# 50011
Sepharose Protein G beads	Invitrogen	Cat# 101242
pHrodo™ Red *E.coli* BioParticles™	Invitrogen	Cat# P35361
Live cell imaging solution	Thermo Fisher Scientific	Cat# A14291DJ
**Antibodies**		
**Primary antibodies**		
Anti-DAP12 (human)	In house*	DAP12.1 and DAP12.2*
Anti-DAP12 (human and mouse)	Cell Signaling Technology	Cat# 12492 Clone: D7G1X
Anti-TREM2 (human)	R&D Systems	Cat# AF1828 or AF18281
Anti-TREM2 (human)	In house [36]	Clone: 4B2A3
Anti-TREM2 (human)	Ch. Haass lab [37]	Clone: 9D11
Anti-Calnexin (human, mouse, rat and etc.)	Millipore	Cat# AB2301
Anti-Calnexin (human)	Abcam	Cat# ab112995 Clone: 6F12BE10
Anti-Ubiquitinylated proteins	Millipore	Cat# 04-263 Clone: FK2
Anti-Actin	Sigma-Aldrich	Cat# A1978 Clone: AC-15
Anti-Glycerinaldehyd-3-phosphat-Dehydrogenase (GAPDH) (human)	Santa Cruz	Cat# sc-47724 Clone: 0411
Anti-Amyloid-Precursor-Protein (APP) (human, mouse and rat)	Biolegend	Cat# BLD-802,801 Clone: C1/6.1
Anti-Alpha 1 Sodium Potassium ATPase (human, mouse, rat and etc.)	Abcam	Cat# ab7671 Clone: 464.6
Anti-Giantin	Abcam	Cat# ab37266 Clone: 9B6
Anti-ERGIC53 (LMAN1)	Thermo Fisher Scientific	Cat# MA5-25345 Clone: OTI1A8
Anti-RER1 (human, mouse and rat)	Sigma-Aldrich	Cat# R4407
Anti-RER1 (human)	Mybiosource	Cat# MBS418574
Anti-spleen tyrosine kinase (SYK) (human)	Cell Signaling Technology	Cat# 80460 Clone:4D10
Anti-Phospho-SYK (Tyr525/526) (human)	Cell Signaling Technology	Cat# 2710 Clone: C87C1
**Secondary antibodies**		
IRDye680 RD donkey anti rabbit	Li-COR	Cat# 926-68073
IRDye800 CW donkey anti goat	Li-COR	Cat# 926-32214
IRDye800CW donkey anti mouse	Li-COR	Cat# 926-32212
IRDye680 RD donkey anti mouse	Li-COR	Cat# 926-68072
IRDye800CW goat anti rat	Li-COR	Cat# 926-32219
Alexa Fluor 546-conjugated anti-rabbit	Invitrogen	Cat# A10040
Alexa Fluor 488-conjugated Anti-mouse	Invitrogen	Cat# A21202
Horseradish peroxidase conjugated goat anti-rat	Rockland	Cat# 612-103-120
Horseradish peroxidase conjugated rabbit anti-goat	Sigma-Aldrich	Cat# A-5420

*The anti-DAP12 antibodies DAP12.1 and DAP12.2 were generated by GenScript Biotech Corporation (USA) by inoculation of rabbits with the peptide CITETESPYQELQGQ and CDVYSDLNTQRPYYK, respectively

### Cell culture

Human embryonic kidney (HEK) 293 cells were cultured in Dulbecco’s Modified Eagle Medium (DMEM, Gibco, USA), supplemented with 10% heat-inactivated Fetal Bovine Serum (FBS, PAN-Biotech, P30-3306, Germany), 1% penicillin/streptomycin (P/S, Gibco, 15140-122, USA) in an atmosphere containing 5% CO_2_ at 37 ℃. Cells were split when the confluence reached 80–90%. For detaching, cells were washed with prewarmed phosphate buffer saline (PBS), followed by incubation with PBS containing 0.05% trypsin and 0.02% EDTA. The process was stopped by resuspending the cells in fresh DMEM supplemented with 10% FBS and 1% P/S. Aliquots of the cell suspension were transferred to new dishes containing the appropriate cell culture medium. Plasmid transfected HEK293 Flp-In cells were cultured in DMEM supplemented with 200 µg/mL Hygromycin B (InvivoGen, ant-hg-5, USA).

THP-1 cells were cultured in T75 flasks with RPMI 1640 medium (Gibco, USA), supplemented with 10% FBS, 1% P/S, 0.05 µM β-Mercaptoethanol (Sigma-Aldrich, M7522, Germany) in a cell incubator with 5% CO_2_ at 37 ℃. Cells were split before the cell concentration reached 1,000,000 cells/mL. THP-1 cells were differentiated into macrophage-like cells by incubation with 5 ng/mL phorbol 12-myristate 13-acetate (PMA, Sigma-Aldrich, P8139, Germany) for 48 h and allowed to recover for 1 day in RPMI 1640 containing 10% FBS, 1% P/S, 0.05 µM β-Mercaptoethanol medium without PMA before further experimental use [38].

### Cell transfection

Five µg of the respective DNA plasmid was mixed with 200 µL Opti-MEM Reduced Serum medium (Gibco, USA). Lipofectamine 2000 (7.5 µL; Thermo Fisher Scientific, 11668-019, USA) was mixed with 200 µL Opti-MEM Reduced Serum medium by flicking and incubated for 5 min at room temperature (RT). Lipofectamine 2000 and the respective plasmid constructs were mixed and incubated for 20 min at RT. Meanwhile the cells were washed twice with PBS and kept in culture medium (DMEM) without any supplements. The DNA-Lipofectamine mixtures were dropwise added to cells cultured in 6 cm diameter dishes and incubated for 16 h at 37 °C, 5% CO_2_. For stable transfections of HEK293 Flp-In cells, a co-transfection of 7.2 µg pOG44 (Invitrogen, USA) encoding Flp recombinase and 0.8 µg of the respective cDNA construct in a pcDNA5/FRT vector (Invitrogen, USA) was performed. Medium was changed 24 h after transfection, and supplemented with 200 µg/mL Hygromycin B for selection of cells expressing the respective gene of interest [36].

### Generation of TREM2 ko and RER1 ko THP-1 cell lines

CRISPR RNA (crRNA) and transactivating crRNA (tracrRNA, IDT, 1073190, USA) duplex, representing single guide RNAs (sgRNAs) for targeting TREM2 (***GCCATCACAGACGATACCCT***, IDT, Hs.Cas9 TREM2.1.AA, USA) or RER1 (***TGTGCGATGGGTCGTGACAC***, IDT, Hs.Cas9.RER1.1.AA, USA) were designed and obtained from Integrated DNA technologies (IDT, USA). For preparation of RNA oligos, crRNA and tracrRNA were diluted to the same concentration (1 µM) and mixed. The mixed RNAs were heated at 95 ℃ for 5 min and cooled down at RT. Cas9 enzyme (IDT, 1,081,061, USA) was diluted to a working concentration (1 µM) in Opti-MEM Reduced Serum medium. For each well of a 96-well tissue culture plate, 1.5 µL sgRNA (crRNA and tracrRNA duplex, 1 µM) and 1.5 µL Cas9 enzyme (1 µM) were mixed in 22 µL Opti-MEM Reduced Serum medium and incubated at RT for 5 min to assemble the ribonucleoprotein (RNP) complexes. Next, RNP complexes (25 µL) were mixed with lipofectamine RNAiMAX (Invitrogen, 13778-150, USA, 1.2 µL) and Opti-MEM Reduced Serum medium (23.8 µL) and incubated at RT for 20 min to form transfection complexes. Meanwhile, THP-1 cells were washed with PBS and diluted to 400,000 cells/mL in complete media without antibiotics. Afterwards, 50 µL of the transfection solution containing the RNP complexes were transferred into one well of a 96-well plate. Hundred µL of the cell suspension (40,000 cells) were added to yield a final concentration of RNP complexes of 10 nM, and the plate was incubated for 4 h. Single-cell clones were obtained by diluting the cells to 1 cell/100 µL and cultured in 96-well plates. Single-cell clones were further expanded and analyzed for protein expression and DNA sequencing.

### Preparation of cellular membrane fractions

For preparation of cellular membranes, cells were washed once with ice cold PBS, scraped in an appropriate volume of hypotonic buffer (10 mM Tris-HCl, 1 mM EDTA, 1 mM EGTA in dH_2_O, pH 7.6) supplemented with protease inhibitor cocktail (Sigma-Aldrich, 04693116001, Germany) and collected in tubes. Cells were incubated on ice for 10 min and subsequently homogenized using a syringe with a 0.6 mm cannula, drawing 20 times. After centrifugation for 10 min at 200 x g and 4 °C, the resultant supernatant was transferred into a new tube and centrifuged for 1 h at 16,000 x g and 4 °C. The supernatant was removed and the remaining pellet was considered as a crude membrane fraction. For extraction of proteins, the membrane fraction was resuspended in lysis buffer (1% n-dodecyl-β-d-Maltoside (Sigma-Aldrich, D4641, Germany), 50 mM Tris–HCl (pH 8.0), 150 mM NaCl, 1 mM EDTA, 1.5 mM MgCl_2_, 10% glycerol) supplemented with protease inhibitor and phosphatase inhibitor cocktail (Sigma-Aldrich, 4906837001, Germany), and incubated for 20 min on ice. The lysate was centrifuged for 10 min at 16,000 x g and 4 °C and the supernatant used either for co-immunoprecipitation or direct analysis by sodium dodecyl-sulfate polyacrylamide gel electrophoresis (SDS-PAGE) and western immunoblotting.

### Sodium dodecyl-sulfate polyacrylamide gel electrophoresis (SDS-PAGE) and western immunoblotting

Samples were mixed with SDS sample buffer with or without dithiothreitol (DTT, 100 mM) as indicated and heated for 5 min at 95 °C. Proteins were separated using pre-cast NuPAGE Novex Bis-Tris Gels 4–12% (Invitrogen, USA), NuPAGE running chambers, and NuPAGE MES SDS Running Buffer (Invitrogen, NP0002, USA) at 150 V. Proteins were transferred onto nitrocellulose membranes by wet transfer technique at a constant current of 400 mA for 1 h and 45 min. After blocking for 1 h with constant agitation in Tris-Buffered Saline, 0.1% Tween® 20 Detergent (TBST) containing 5% milk powder, membranes were incubated over night at 4 °C with primary antibodies diluted in TBST. Membranes were washed 3 times for 5 min, and incubated in TBST containing the respective secondary antibody conjugated either with horseradish peroxidase or a fluorophore (IRDye800CW, 680RD, Li-COR Biosciences, Germany) for 1 h at RT. After washing the membrane 3 times for 5 min, signals were detected using a Chemidoc XRS Imager (Bio-Rad, USA) or Odyssey® CLx (Li-COR Biosciences, Germany). ImageJ (NIH, USA) was used for quantitative analysis of the blots.

### Real-time quantitative reverse transcription PCR (real-time qRT-PCR)

Total RNA was extracted from THP-1 cells by RNeasy® Mini Kit (QIAGEN, 74104, Germany) and genomic DNA was removed using RNase-Free DNase (QIAGEN, 79254, Germany). Single-stranded cDNA was generated from total RNA by reverse transcription using a RevertAid First Strand cDNA Synthesis Kit (Thermo Fisher Scientific, K1622, USA) according to the manufacturer’s instructions. Quantitative PCR was performed using QuantiTect® SYBR® Green PCR Kit (QIAGEN, 204143 or 204163, Germany). The mRNA levels of DAP12 (QIAGEN, QT00077518, Germany) and TREM2 (QIAGEN, QT00063868, Germany) were normalized to Hypoxanthine phosphoribosyltransferase 1 (HPRT1, QIAGEN, QT00059066, Germany) and Ubiquitin C (UBC, accession number M26880, forward ATTTGGGTCGCGGTTCTTG and reverse TGCCTTGACATTCTCGATGGT) expression in each sample.

### Immunocytochemistry

Cells were seeded on poly-L-lysine (PLL, Sigma-Aldrich, P6282, Germany) coated coverslips or ibidi dishes (ibidi, Germany) and fixed with 4% paraformaldehyde (PFA, Sigma-Aldrich, 16005, Germany) in PBS for 20 min. After washing 3 times with PBS, cells were permeabilized in PBS containing 0.1% Triton X-100 (Carl Roth, 3051.2, Germany) for 15 min at RT, followed by blocking in PBS containing 0.1% Tween® 20 (PBST) supplemented with 3% bovine serum albumin (BSA) for 1 h. Cells were incubated with primary antibodies diluted in PBST containing 1% BSA at RT for 1 h and washed 3 times with PBST. Afterwards, cells were incubated with secondary antibodies diluted in PBST containing 1% BSA and 4′,6-diamidino-2-phenylindole (DAPI, Invitrogen, USA) for 1 h at RT and washed 3 times with PBST followed by two washes with PBS. Subsequently, the coverslips were mounted on microscopic slides using Immu-Mount™ (Thermo Fisher Scientific, 9990402, USA). Samples processed on ibidi dishes were mounted using ibidi mounting medium (ibidi, 50011, Germany). Images were taken with a Zeiss microscope (AxioVert 200) supplied with a Zeiss ApoTome using a 63/1.4 objective and EGFP, DsRed and DAPI fluorescence filter sets. Images were processed with ImageJ (NIH, USA).

For quantitative image analysis, images were randomly captured with identical camera settings within individual experiments. Analysis of colocalization was done with the colocalization processing module (Coloc 2) of Fiji ImageJ. A total number of at least 65 cells for each experimental condition was scored by a person blind to the samples.

### Co-immunoprecipitation

Cellular membrane lysates were incubated with 5 µg of the indicated antibodies for 4 h at RT. Afterwards, 20 µL Sepharose Protein G beads (Invitrogen, 101242, USA) were added to the samples and incubated for 1 h at RT. Beads were collected by centrifugation, washed for twice with the buffer (50 mM Tris–HCl (pH 8.0), 150 mM NaCl, 1 mM EDTA, 1.5 mM MgCl_2_, 10% glycerol) and once with PBS. Bound proteins were denatured at 95 °C for 5 min in SDS sample buffer and subjected to western immunoblot analysis.

### AlphaLISA technology

Detection of phospho-SYK (pSYK) in cell lysates was performed according to manufacturer’s instructions (AlphaLISA® SureFire® Ultra™, Perkin Elmer, USA). Briefly, cells were lysed with Lysis Buffer-Ultra (included in the kit, AlphaLISA® SureFire® Ultra™ p-SYK (Tyr525/526) Assay Kit, Perkin Elmer, ALSU-PSYK-A500, USA) for 10 min. Ten µL of cell lysates were transferred into a 384-well Optiplate (Perkin Elmer, 6007680, USA) and incubated with 5 µL acceptor beads prior to 5 µl donor beads for 1 h in the dark, respectively. The luminescence signals were measured using a Tecan Spark reader (Tecan, Switzerland) with standard AlphaLISA settings.

### Phagocytosis

Cells were seeded at a density of 100,000 cells/well into 96-well flat-bottom plates with black wall (Greiner Bio-One, 655090, Germany) or black ibiTreat 96-well µ-plates (ibidi, 89626, Germany) and were cultured for 48 h in complete RPMI 1640 medium (10% FBS, 1% P/S) in presence of 5 ng/mL of PMA and recovered in normal medium for 1 day without PMA. On the day of the experiment, cells were pre-incubated with or without 10 µM cytochalasin D (cyto D, Sigma-Aldrich, C8273, Germany) for 30 min. Meanwhile, equal volumes of pHrodo™ Red *E.coli* BioParticles™ (1 mg/mL, Invitrogen, P35361, USA) and live cell imaging solution (Thermo Fisher Scientific, A14291DJ, USA) were mixed with or without 10 µM cyto D. The pre-incubation medium was removed from the cells, and 100 µL of the mixture solution was added. Fluorescence was analyzed using an infinite M200Pro reader (Tecan, Switzerland) at the indicated time points. Fluorescence signals at 568 nm as well as bright field images were taken using a Keyence BZ-X800 microscope with a 40/0.60 objective, and processed using ImageJ (NIH, USA). For the quantification of phagocytosis, pHrodo fluorescence intensity of samples incubated with cyto D was subtracted.

### Statistical analysis

Data were analyzed using GraphPad Prism 9 (GraphPad Software, Inc, USA). All data were tested for normality and subsequently analyzed by one-way ANOVA followed by post hoc Tukey’s multiple comparisons test or Student’s t-test (unpaired, two-tailed). A p-value less than 0.05 was considered as statistically significant (*p < 0.05; **p < 0.01; ***p < 0.001; ****p < 0.0001). Information on the number of experiments and replicates is provided in the figure legends.

## Results

### Deletion of TREM2 increases degradation of DAP12 by the proteasome

**Fig. 1:**
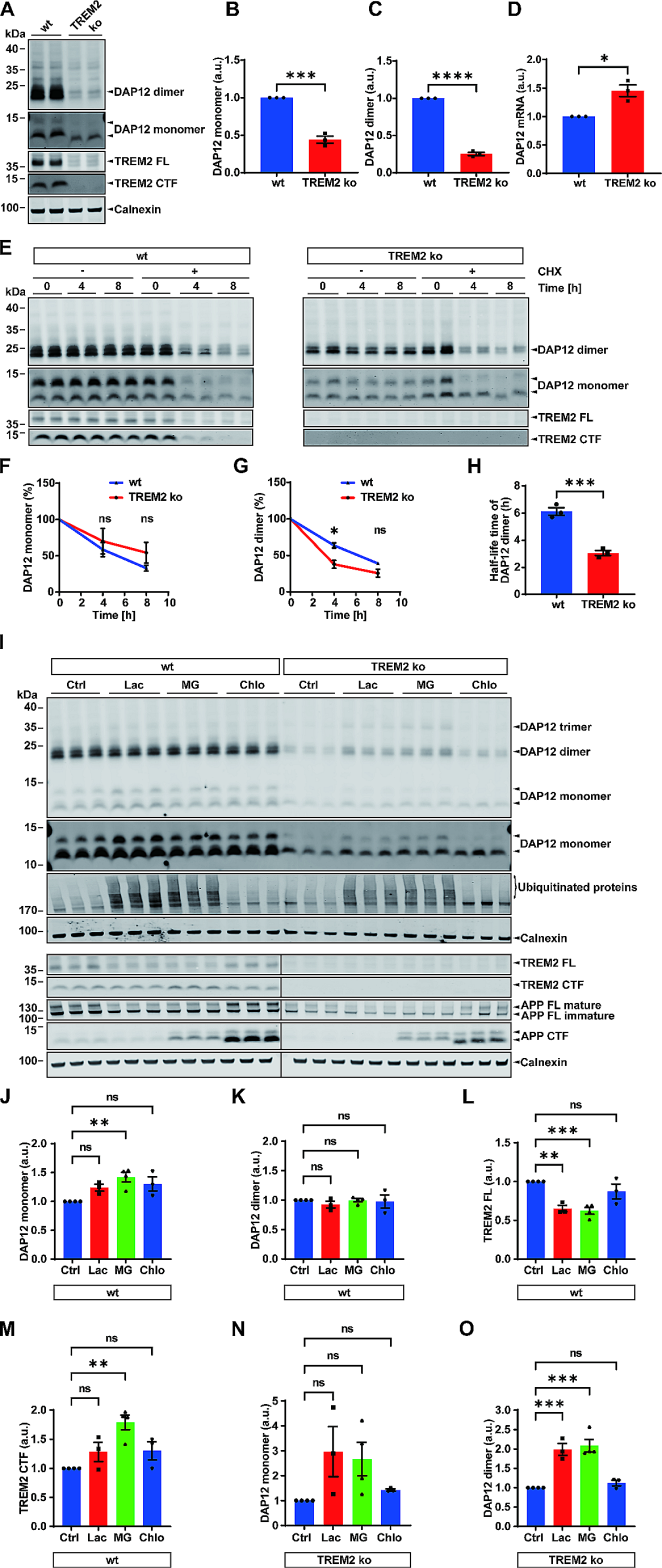
Increased turnover of DAP12 in the absence of TREM2. (**A**) Detection of DAP12 in macrophage-like differentiated THP-1 wt and TREM2 knockout (ko) cells. Cellular membranes were isolated and the indicated proteins analyzed by western immunoblotting. TREM2 FL: TREM2 full length protein; TREM2 CTF: TREM2 C-terminal fragment. (**B**) and (**C**) Quantification of monomeric and dimeric DAP12 by western immunoblotting (as shown in **A**). Monomeric and dimeric DAP12 was normalized to calnexin present in the membrane fraction. Mean ± SEM of three independent experiments each performed with duplicate or triplicate samples. Each data point represents the mean value of an individual experiment. Student’s t-test (unpaired, two-tailed). ***p < 0.001, ****p < 0.0001. (**D**) Comparison of the DAP12 mRNA levels in differentiated THP-1 wt andTREM2 ko cells by real-time qRT-PCR. Values represent Mean ± SEM of three independent experiments. Each data point represents the mean value of an individual experiment. Student’s t-test (unpaired, two-tailed). *p < 0.05. (**E**) Analysis of DAP12 stability in THP-1 wt and TREM2 ko cells. THP-1 differentiated macrophage-like wt and TREM2 ko cells were incubated without (Ctrl) or with cycloheximide (CHX, 100 µg/mL) for the indicated time points. Cellular membranes were isolated and the indicated proteins were analyzed by western immunoblotting. (**F**) - (**H**) Quantification of DAP12 turnover upon cell treatment with CHX. The level of DAP12 was normalized against the level of DAP12 in non-treated control cells at corresponding time points. The half-life time of DAP12 dimer was about 6.12 h in wt cells, and 3.06 h in TREM2 ko cells. Mean ± SEM of three independent experiments each performed with duplicate samples. Each data point represents the mean value of an individual experiment. Student’s t-test (unpaired, two-tailed). *p < 0.05. The statistical differences marked with the asterisk (*) and no significance (ns) are between wt and TREM2 ko cells at 4 h and 8 h, respectively. (**I**) Characterization of degradation pathways for DAP12 and TREM2. THP-1 wt and TREM2 ko cells were incubated without (Ctrl) or with MG132 (MG, 10 µM), lactacystin (Lac, 10 µM), chloroquine (Chlo, 50 µM) for 4 h. Cellular membranes were isolated and the indicated proteins detected by western immunoblotting. Ubiquitinated proteins and amyloid precursor protein (APP) were detected as positive controls for efficient inhibition of proteasomal and lysosomal activity, respectively. The second gel image panel from the top is a section of the upper image with higher intensity to better visualize DAP12 monomers. TREM2 FL: TREM2 full length protein; TREM2 CTF: TREM2 C-terminal fragment; APP FL: APP full length protein; APP CTF: APP C-terminal fragment. (**J**) - (**O**) Quantification of DAP12 monomer (**J**, **N**), dimer (**K**, **O**), and TREM2 FL (**L**) and CTF (**M**) in THP-1 wt (**J** - **M**) and TREM2 ko (**N**, **O**) cells. Indicated proteins were normalized to the loading control protein calnexin. Data represent the Mean ± SEM of three or four independent experiments each performed with duplicate or triplicate samples. Each data point represents the mean value of an individual experiment. One way ANOVA (post hoc Tukey’s multiple comparisons test). *p < 0.05, **p < 0.01, ***p < 0.001

In order to investigate the metabolism of endogenous DAP12 and TREM2, we used human THP-1 cells that could be differentiated into a macrophage-like phenotype by treatment with PMA [39–41]. Differentiation with PMA strongly increased expression of DAP12 and TREM2 as demonstrated by elevated mRNA and protein levels (Suppl. Figure S1A-C). SDS-PAGE analysis under non-reducing conditions revealed that DAP12 predominantly exists in dimeric form (Suppl. Figure S1A). The low levels of monomeric DAP12 might represent a pool of DAP12 before dimerization. As expected, DAP12 dimers dissociated to monomers under reducing conditions (Suppl. Figure S1A). However, the monomers derived from dissociated DAP12 dimers under reducing conditions showed slower migration in SDS gels as compared to the monomeric DAP12 pool detected under non-reducing conditions (Suppl. Figure S1). It remains to be determined whether the different migration of monomers is due to altered glycosylation or phosphorylation states of DAP12 before and after dimerization. Interestingly, levels of dimeric and monomeric DAP12 were significantly decreased in TREM2 ko cells (Fig. 1A-C). However, the level of DAP12 mRNA was even slightly increased in the TREM2 ko cells compared with wt cells (Fig. 1D). Chase assays revealed increased degradation of DAP12 dimers in TREM2 ko cells as compared to TREM2 expressing cells. The half-life time of DAP12 dimers in wt cells was about 6.12 h, while it was about 3.06 h in TREM2 ko cells (Fig. 1E, G, H). However, the turnover of the monomeric DAP12 pool was not significantly affected by TREM2 deficiency (Fig. 1F).

Next, we wanted to characterize the degradation systems involved in DAP12 turn-over. There are two major pathways for protein degradation, the ubiquitin-proteasome pathway, which mainly degrades short-lived proteins and soluble misfolded proteins [42, 43], and the autophagy-lysosomal pathway, which is responsible for degradation of entire organelles, protein aggregates, and cargo in cytoplasmic vesicular compartments [44]. In these experiments, lactacystin and MG132 were used as proteasome inhibitors, while lysosomal activity was inhibited by chloroquine (Fig. 1I). After 4 h treatment, lactacystin and MG132 had little if any effect on DAP12 dimer levels in THP-1 wt cells (Fig. 1K). In contrast, both proteasomal inhibitors strongly elevated levels of dimeric DAP12 in TREM2 ko cells, indicating that TREM2 deficiency caused increased proteasomal degradation of DAP12 dimers (Fig. 1O). Inhibition of proteasomal activity also slightly increased levels of monomeric DAP12 in both wt and TREM2 ko cells (Fig. 1J and N). Notably, proteasomal inhibition also led to the accumulation of DAP12 trimers, particularly pronounced in TREM2 ko cells, albeit at a much lower level as compared to dimeric DAP12 (Fig. 1I). Chloroquine had no effect on DAP12 levels in either genotype (Fig. 1J, K, N, O). The treatment of THP-1 wt cells with lactacystin and MG132 decreased levels of TREM2 full length protein (TREM2 FL, Fig. 1L). In contrast, MG132 rather increased levels of TREM2  C-terminal fragments (TREM2 CTFs) (Fig. 1M). Cell treatment with lactacystin also tended to increase levels of TREM2 CTFs, but this effect was not statistically significant. TREM2 CTFs derive from the full-length protein by ectodomain shedding and remain inserted into cellular membranes [31, 45]. The inhibition of lysosomal activity by chloroquine had no significant effect on TREM2 CTFs (Fig. 1M), suggesting that the degradation of TREM2 CTFs rather involves proteasomal activity than lysosomal activity. To prove that chloroquine indeed inhibited lysosomal activity under our experimental conditions, we detected APP CTFs that are known to be partially degraded by lysosomes [46–48]. Consistent with previous findings [49, 50], APP CTFs accumulated upon cell treatment with chloroquine. Together, these data demonstrate destabilization of DAP12 in the absence of TREM2 involving increased degradation by the proteasome. Proteasomal activity could also contribute to the degradation of TREM2 CTFs.

To further investigate the effects of TREM2 on DAP12 metabolism and subcellular transport without potential interference by other immunoreceptors that might interact with DAP12, we chose HEK293 Flp-In cells that allow expression of cDNAs from a defined genetic locus [51]. This model was applied previously to study TREM2-DAP12 expression and signaling [31, 36]. We generated cell clones that stably express a bicistronic cDNA construct encoding TREM2 and DAP12 separated by a T2A linker sequence to ensure stoichiometric expression of both proteins. In addition, stable clones were generated expressing DAP12 alone or together with the NHD-associated TREM2 K186N mutation. This mutation is known to prevent the interaction with DAP12 [27, 30]. Indeed, co-immunoprecipitation proved the lack of interaction of the TREM2 K186N mutant with DAP12 (Suppl. Figure S2 A).

**Fig. 2:**
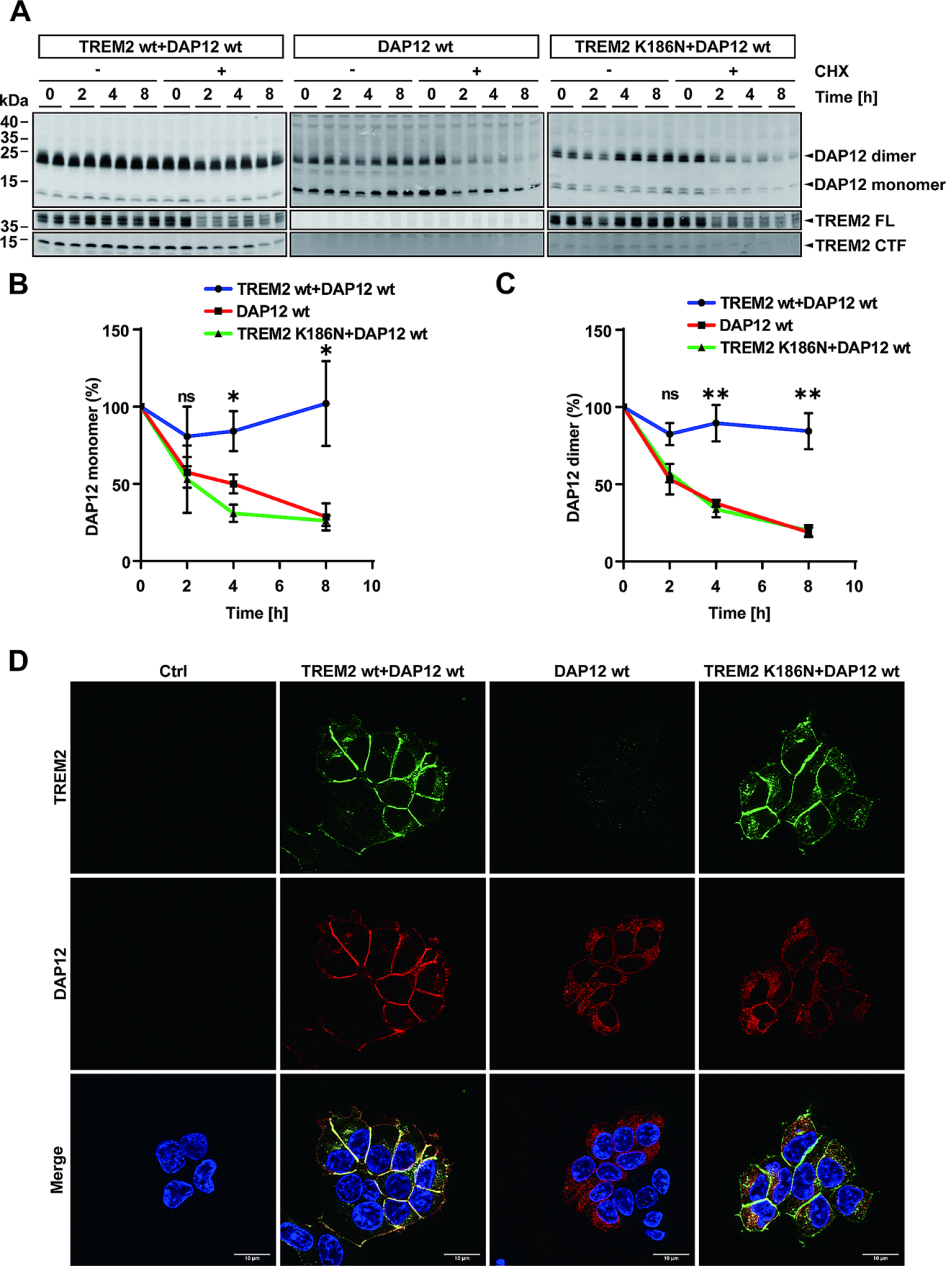
Absence of TREM2 or the disease-associated TREM2 K186N destabilizes DAP12 and reduces its expression at the cell surface. (**A**) HEK293 Flp-In cells stably overexpressing the indicated proteins were incubated without (Ctrl) or with cycloheximide (CHX, 100 µg/mL) for the indicated time points. Cellular membranes were isolated. DAP12 and TREM2 were detected by western immunoblotting. TREM2 FL: TREM2 full length protein; TREM2 CTF: TREM2 C-terminal fragment. (**B**) - (**C**) Quantification of DAP12 levels at the indicated time periods upon cell treatment with CHX (t = 0 h was set as 100%). The level of DAP12 was normalized against the level of DAP12 in non-treated control cells at the corresponding time points. Data represent the Mean ± SEM of three independent experiments each performed with duplicate samples. One-way ANOVA (post hoc Tukey’s multiple comparisons test). Statistical significance of differences at the individual time points is indicated by asterisks (*p < 0.05; **p < 0.01; ns, no significance). No significant difference was observed between cells overexpressing DAP12 only or together with the TREM2 K186N mutant. (**D**) Immunocytochemical detection of DAP12 and TREM2 in HEK293 Flp-In cells stably expressing the indicated proteins. Representative images are shown. TREM2 (green) and DAP12 (red) were detected with antibodies 4B2A3 and D7G1X, respectively, and appropriate secondary antibodies as described in the Method section. Nuclei were stained with DAPI. Scale bar = 10 μm

Protein chase experiments revealed strongly increased degradation of DAP12 dimers and monomers in cells expressing DAP12 alone or together with the TREM2 K186N mutant (Fig. 2A-C) as compared to cells that co-express TREM2 wt, thereby verifying the results obtained with the THP-1 cell model. In line with the results obtained with THP-1 cells, levels of monomeric DAP12 were much lower as compared to that of dimeric DAP12 in the HEK293 cell model with the overexpression of TREM2 and DAP12 variants (Suppl. Figure S3). The inhibition of proteasomal activity significantly increased levels of dimeric DAP12 in HEK293 cells expressing DAP12 alone or together with the TREM2 K186N mutant, but not in cells co-expressing TREM2 wt (Suppl. Figure S3 C, G, I). Levels of monomeric DAP12 were significantly affected by proteasome inhibition only in cells co-expressing the TREM2 K186N mutant (Suppl. Figure S3B, F, H). In these cells, DAP12 trimers were also detected upon treatment with proteasome inhibitors (Suppl. Figure S3 A).

### Retention of unassembled DAP12 in early secretory compartments

Next, we analyzed the subcellular localization of TREM2 and DAP12 by immunocytochemistry. In TREM2 wt-DAP12 wt expressing HEK293 Flp-In cells, DAP12 localized mainly at the plasma membrane together with TREM2 (Fig. 2D). In contrast, in cells expressing DAP12 together with the TREM2 K186N, these proteins showed differential localization. While the TREM2 K186N was still detected predominantly at the cell surface, DAP12 mainly localized in intracellular vesicles with little if any expression at the plasma membrane (Fig. 2D). DAP12 also showed predominant localization to intracellular vesicles when expressed without TREM2. These data indicate that the interaction with TREM2 not only stabilizes DAP12, but is also required for efficient transport of DAP12 to the cell surface.

**Fig. 3:**
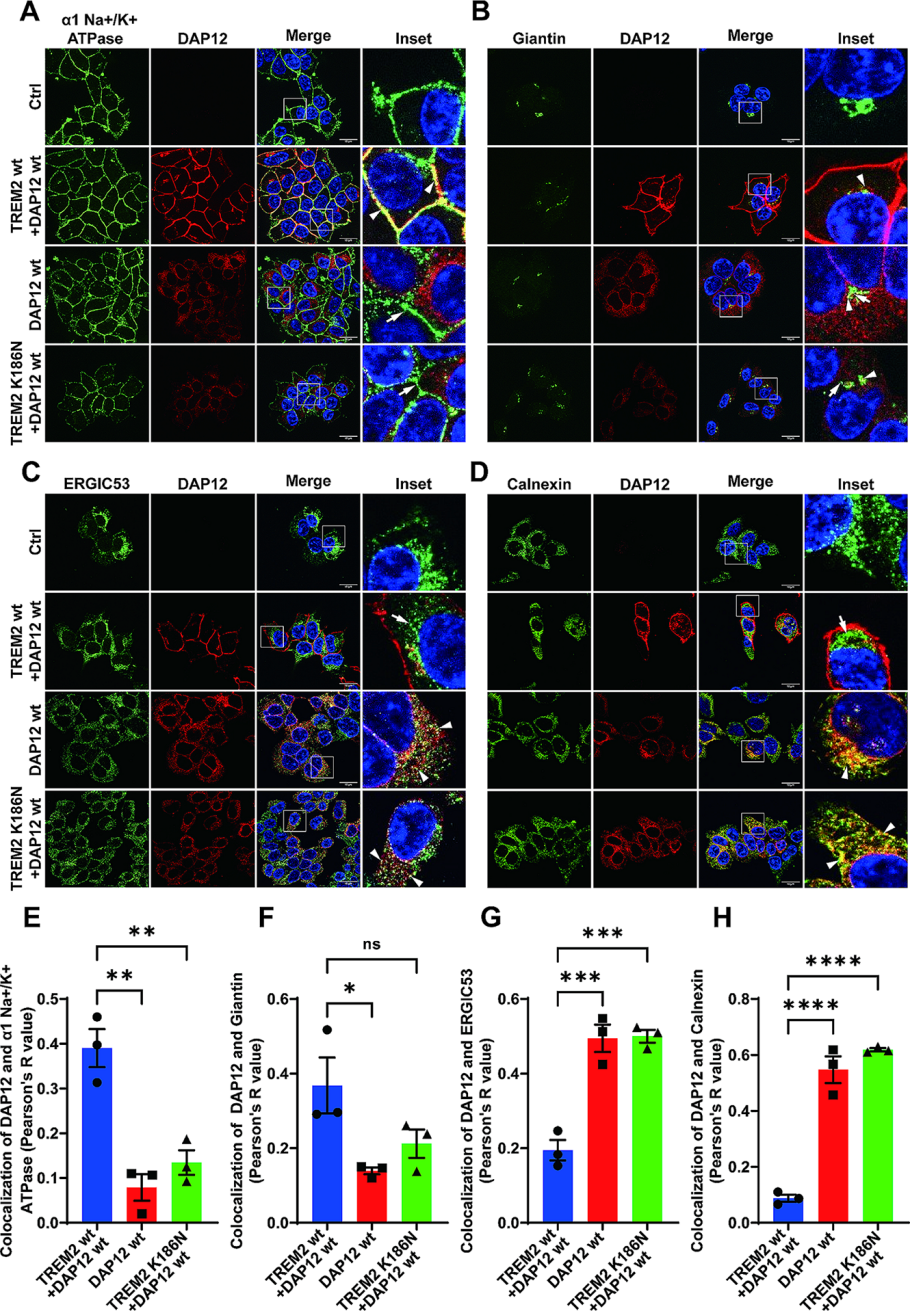
DAP12 accumulates in early secretory compartments in the absence of TREM2 interaction. (**A**) - (**D**) Immunocytochemical detection of DAP12 in HEK293 Flp-In cells stably overexpressing DAP12 only (DAP12) or together with TREM2 (TREM2 + DAP12) or the TREM2 K186N mutant (TREM2 K186N + DAP12). Representative images are shown. Cells were co-stained for DAP12 (red) and the indicated marker proteins. Nuclei were counterstained with DAPI. Arrowheads indicate colocalization of DAP12 with the respective marker protein. Arrows indicate separate localization of DAP12 from the respective marker protein. Scale bar = 10 μm. (**E**) - (**H**) Pearson’s R value of colocalization of DAP12 with the respective maker proteins. Values represent Mean ± SEM of three independent experiments. Each data point represents the mean value of an individual experiment. One way ANOVA (post hoc Tukey’s multiple comparisons test). *p < 0.05, **p < 0.01, ***p < 0.001, ****p < 0.0001

The subcellular localization of DAP12 was analyzed in more detail by co-staining with several marker proteins of distinct subcellular compartments. First, co-staining of DAP12 and alpha 1 sodium potassium ATPase (α1 Na+/K + ATPase) confirmed localization of DAP12 at the cell surface in TREM2 wt-DAP12 wt expressing cells. The colocalization of DAP12 and α1 Na+/K + ATPase was significantly reduced in cells that express DAP12 wt alone or together with the TREM2 K186N mutant (Fig. 3A and E). DAP12 also colocalized with Giantin in TREM2 wt-DAP12 wt expressing cells, a marker for the Golgi compartment (Fig. 3B and F). The colocalization of DAP12 and Giantin was significantly lower in cells not co-expressing TREM2 (Fig. 3F). The colocalization of DAP12 and Giantin also slightly decreased in TREM2 K186N-DAP12 wt expressing cells as compared to TREM2 wt-DAP12 wt expressing cells, although the difference was not statistically significant (Fig. 3F). More importantly, in cells expressing DAP12 alone or together with the TREM2 K186N mutant, DAP12 showed increased colocalization with calnexin (Fig. 3D and H) and ERGIC53 (Fig. 3C and G), two marker proteins for the ER and the ER Golgi intermediate compartment, respectively. Together, these data indicate retention of DAP12 in (and/or retrieval of DAP12 to) compartments of the early secretory pathway when the protein is not incorporated into a complex with TREM2.

### The aspartic acid residue within the transmembrane domain is critical for retention of unassembled DAP12 in early secretory compartments

**Fig. 4:**
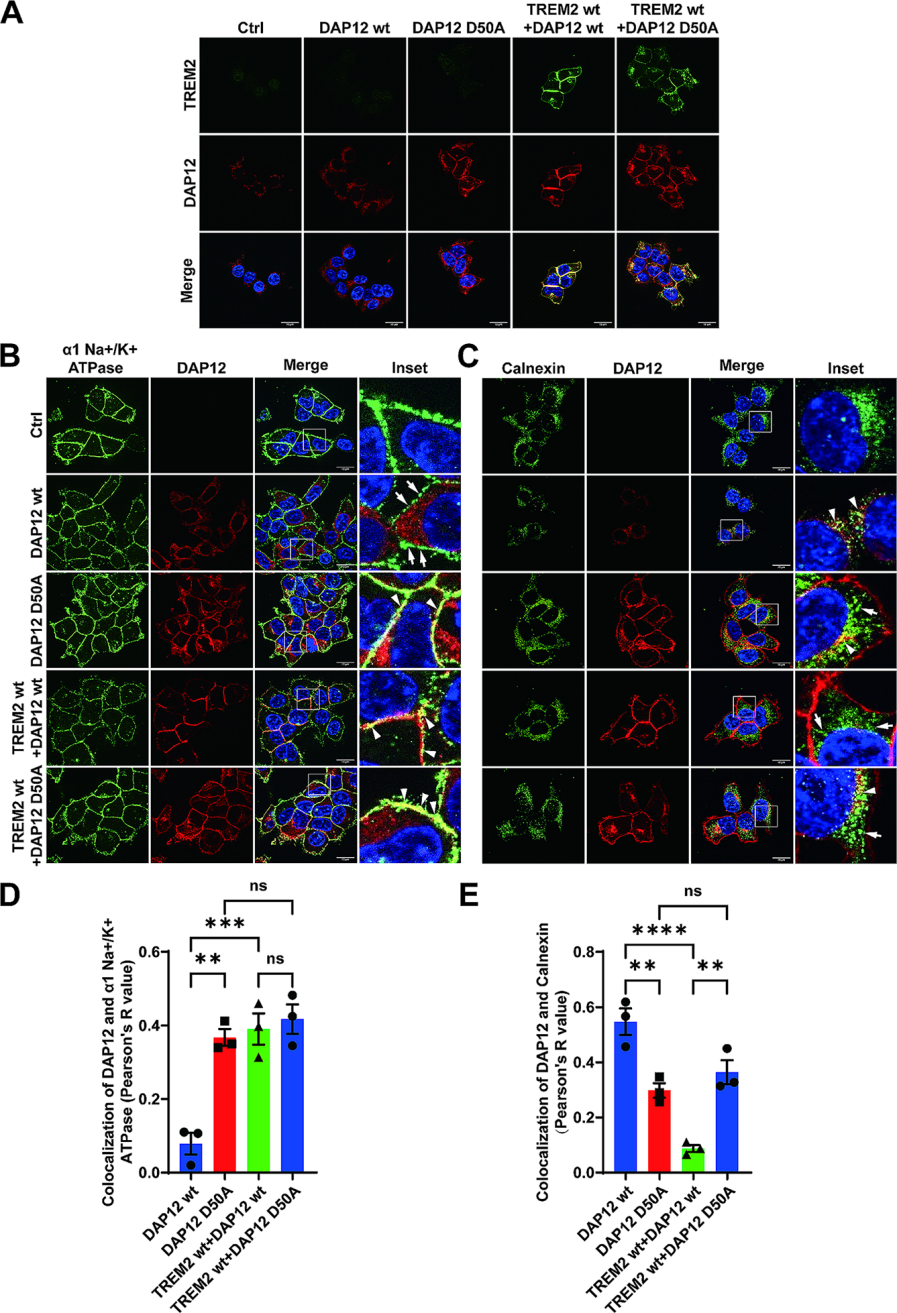
Critical role of D50 within the transmembrane domain of DAP12 for the retention in early secretory compartments. (**A**) Immunocytochemical detection of DAP12 in HEK293 Flp-In cells stably overexpressing DAP12 wt or DAP12 D50A with or without TREM2 wt. Non-transfected HEK293 Flp-In cells served as control (Ctrl). Representative images are shown. TREM2 (detected by antibody 4B2A3) is shown in green and DAP12 (detected by antibody D7G1X) in red. Nuclei were counterstained with DAPI. Scale bar = 10 μm. (**B**) - (**C**) Immunocytochemical detection of DAP12 and TREM2 in HEK293 Flp-In cells stably overexpressing the indicated DAP12 variant with or without TREM2. Non-transfected HEK293 Flp-In cells (Ctrl) served as control. Shown are representative images. Cells were co-stained for DAP12 (red) and the indicated marker proteins (green). Nuclei were counterstained with DAPI. Arrowheads indicate colocalization of DAP12 with the respective marker protein. Arrows indicate separate localization of DAP12 from the respective marker protein. Scale bar = 10 μm. The colocalization of DAP12 with giantin (cis- and medial-Golgi network) and ERGIC53 (ER Golgi intermediate compartment) is shown in Suppl. Figure S4 A-B. (**D**) - (**E**) Pearson’s R value of colocalization of DAP12 with the respective maker proteins. Values represent Mean ± SEM of three independent experiments. Each data point represents the mean value of an individual experiment. One way ANOVA (post hoc Tukey’s multiple comparisons test). **p < 0.01, ***p < 0.001, ****p < 0.0001. The quantification of colocalization of DAP12 with giantin or ERGIC53 is shown in Suppl. Figure S4 C-D

Previous studies found that polar amino acid residues in TMDs could mediate the retention of select proteins in or retrieval to the ER [52]. To explore whether the single aspartic acid residue (D) at position 50 in the TMD of DAP12 is potentially responsible for the retention of unassembled DAP12, this residue was mutated to an alanine residue (A). It has been shown previously that this mutation prevents electrostatic interaction, and thus, the complex formation with TREM2 [7, 29]. This effect was verified by coimmunoprecipitation assays (Suppl. Figure S2B). In a previous study using Ba/F3 cells, it was shown that overexpressed DAP12 D50A, but not DAP12 wt could be detected at the cell surface by flow cytometry, suggesting that the D50 residue inhibits the transport of DAP12 to the plasma membrane [3]. Thus, we analyzed the subcellular localization of DAP12 wt and the DAP12 D50A mutant in the presence or absence of TREM2. Interestingly, in cells expressing DAP12 D50A alone or together with TREM2 wt, DAP12 D50A was detected at the plasma membrane and in the Golgi compartment as indicated by the colocalization with alpha 1 sodium potassium ATPase (α1 Na+/K + ATPase) (Fig. 4B and D) and with Giantin (Suppl. Figure S4 A and C), respectively. The colocalization of DAP12 D50A with α1 Na+/K + ATPase (Fig. 4B and D) or with Giantin (Suppl. Figure S4 A and C) was very similar as that for DAP12 wt when co-expressed with TREM2 wt, and significantly higher than that of DAP12 wt in the absence of TREM2 (Suppl. Figure S4 A and C). Moreover, the colocalization of DAP12 D50A with calnexin was reduced as compared to that of the DAP12 wt protein in the absence or presence of TREM2 (Fig. 4C and E). The colocalization of DAP12 D50A and ERGIC53 also tended to be lower compared to that of the DAP12 wt protein, but this difference was not statistically significant (Suppl. Figure S4B and D). As DAP12 D50A does not interact with TREM2, it can also be ruled out that TREM2 wt (when co-expressed) assisted in the transport of this DAP12 mutant in the secretory pathway. Together, these data indicate that the negatively charged D50 residue in the transmembrane domain plays a crucial role in the retention of DAP12 in early secretory compartments in the absence of proper interaction with TREM2.

**Fig. 5:**
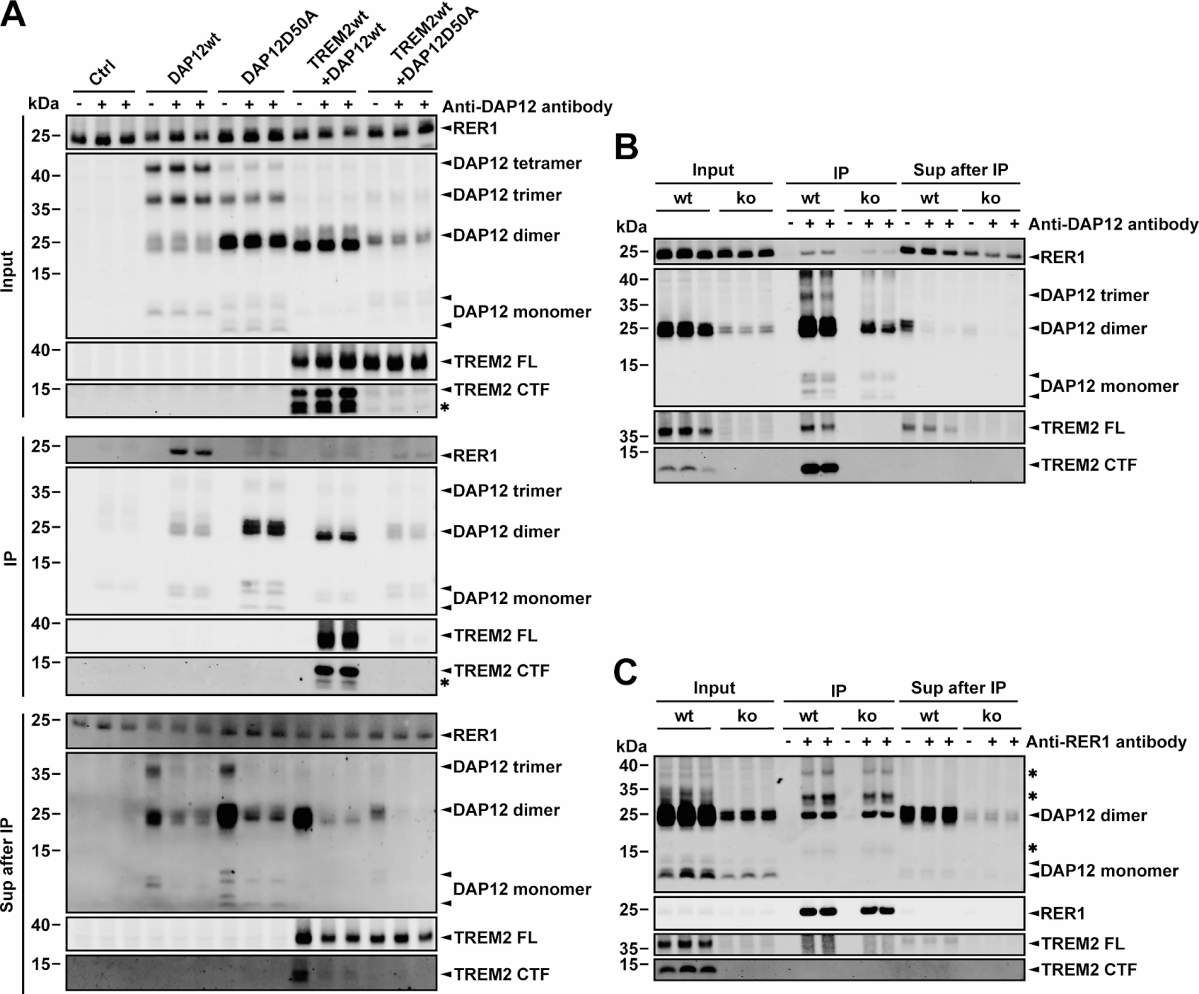
Interaction of DAP12 with RER1. (**A**) - (**C**) HEK293 cells overexpressing the indicated proteins (**A**) or macrophage-like differentiated THP-1 wt and TREM2 ko cells (**B** and **C**) were subjected to co-immunoprecipitation as described in the Methods section. The indicated proteins in the immunoprecipitate (IP) and the remaining supernatant (Sup after IP) were detected by western immunoblotting. The ‘Input’ represents an aliquot of the cell lysate before IP containing 20 µg total protein. Three independent experiments were performed. TREM2 FL: TREM2 full length protein; TREM2 CTF: TREM2 C-terminal fragment; ko: TREM2 ko. The asterisks in A indicate an unidentified band, probably representing a C-terminal degradation product of TREM2 detected by antibody 9D11 that recognizes the intracellular domain of TREM2. The bands marked with asterisks (*) in C are unspecific signals from the anti-RER1 antibody which was used in the IP

Retention of transmembrane proteins, in particular of some components of multiprotein complexes, can be mediated by RER1 that recognizes polar amino acids within their TMDs [33, 53]. Thus, the potential interaction of RER1 with DAP12 was first tested by co-immunoprecipitation from HEK293 cells transiently expressing DAP12 wt or DAP12 D50A variants with or without TREM2 (Fig. 5A). Importantly, DAP12 wt, but not the DAP12 D50A mutant, efficiently co-precipitated with RER1 in the absence of TREM2. However, the co-precipitation of DAP12 wt with RER1 was strongly reduced in the presence of TREM2. These data indicate that RER1 recognizes the polar D50 residue of DAP12 wt when it is not interacting with TREM2. We also noticed that DAP12 wt, but not DAP12 D50A, formed increased levels of SDS resistant trimers and tetramers when expressed in the absence of TREM2. A similar observation was made previously, and it had been proposed that DAP12 interacting immunoreceptors compete with additional DAP12 molecules for interaction with covalently linked DAP12 dimers during complex assembly [28]. However, further work is required to dissect whether oligomerization of DAP12 in the absence of TREM2 modulates the interaction with RER1. The interaction of DAP12 and RER1 was also confirmed in macrophage-like differentiated THP-1 cells endogenously expressing both proteins (Fig. 5B and C).

### RER1 deletion decreases levels of DAP12 and TREM2 and impairs phagocytosis

**Fig. 6:**
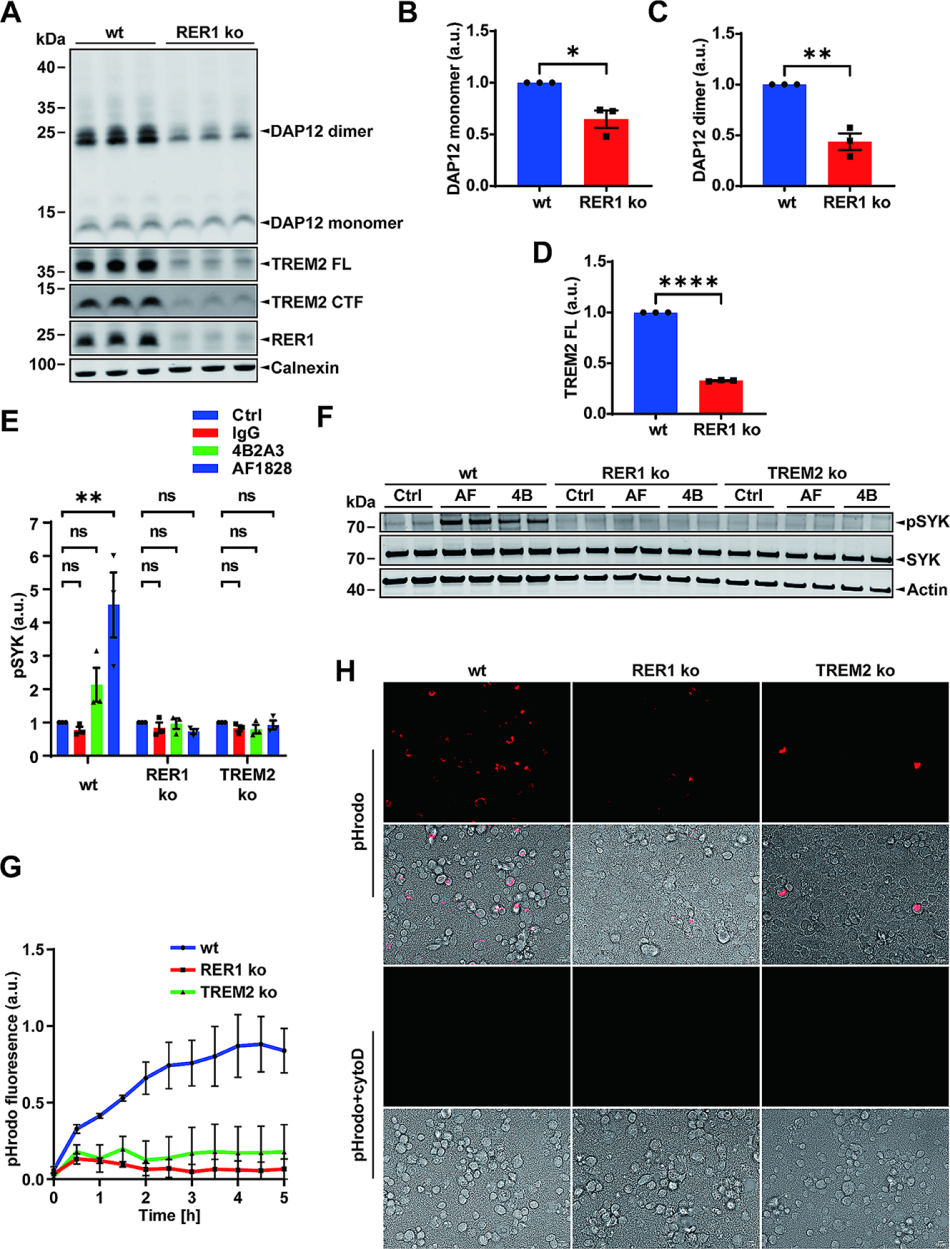
Deletion of RER1 impairs TREM2-DAP12 signaling and phagocytosis. (**A**) Western immunoblotting analysis of the indicated proteins in purified membranes from macrophage-like differentiated THP-1 wt and RER1 ko cells. TREM2 FL: TREM2 full length protein. TREM2 CTF: TREM2 C-terminal fragment. (**B**) - (**D**) Quantification of DAP12 and TREM2 FL expression by western immunoblotting. (B) DAP12 monomer; (**C**) DAP12 dimer; (**D**) TREM2 FL. Levels of the indicated proteins were normalized to levels of calnexin present in the membrane fraction. Data represent the Mean ± SEM of three independent experiments each performed with triplicate samples. Each data point represents the mean value of an individual experiment. Student’s t-test (unpaired, two-tailed). *p < 0.05, **p < 0.01, ****p < 0.0001. (**E**) Differential activation of TREM2-DAP12 signaling in THP-1 wt and RER1 ko cells. Macrophage-like differentiated THP-1 cells were incubated with or without 10 µg/mL of the respective anti-TREM2 antibody (4B2A3 or AF1828) for 10 min. Treatment with an isotype control antibody (IgG) served as a negative control, and THP-1 TREM2 ko cells served as additional controls. After treatment, cells were lysed (whole cell lysates) and phosphorylated SYK (pSYK) detected by AlphaLISA technology. Data represent the Mean ± SEM of three independent experiments each performed with quadruplicate samples. Each data point represents the mean value of an individual experiment. One-way ANOVA (post hoc Tukey’s multiple comparisons test). **p < 0.01. (**F**) Detection of SYK and pSYK in lysates of the indicated cells by western immunoblotting. AF: anti-TREM2 antibody AF1828; 4B: anti-TREM2 antibody 4B2A3. (**G**) Phagocytosis of *E. coli* particles by THP-1 wt and RER1 ko cells. THP-1 wt and RER1 ko cells were seeded in 96-well plates and differentiated to macrophage-like cells by incubation with PMA as described in the Methods section. pHrodo™ Red *E.coli* BioParticles™ were added to the cells and fluorescence measured over time using an Infinite M200Pro reader for 5 h. Cells pre-treated with cytochalasin D (CytoD, 10 µM) for 30 min served as control. Data represent the Mean ± SEM of two independent experiments each performed with triplicate samples. (**H**) Representative images of THP-1 wt and RER1 ko cells from the phagocytosis assay (as described in G) were taken at 2 h after addition of pHrodo™ Red *E.coli* BioParticles™. Scale bar = 10 μm

To further explore the role of RER1 in TREM2-DAP12 assembly, we knocked out the RER1 gene in THP-1 cells. Surprisingly, the deletion of RER1 led to decreased levels of both, DAP12 and TREM2 (Fig. 6A-D). The strong decrease of both proteins was not associated with significant changes of DAP12 and TREM2 mRNA levels, although there was a trend to decreased TREM2 mRNA expression (Suppl. Figure S5B and C). Notably, proteasomal inhibition (MG132 treatment) partially stabilized DAP12 dimer in RER1 ko cells (Suppl. Figure S5D-H). However, additional posttranscriptional and/or posttranslational mechanisms could contribute to the strong decrease of DAP12 and TREM2 proteins in RER1 ko cells.

TREM2-DAP12 signaling can be specifically stimulated by cross-linking anti-TREM2 antibodies [36, 54, 55]. Therefore, the monoclonal 4B2A3 and the polyclonal AF1828 anti-TREM2 antibodies were used to specifically stimulate TREM2-DAP12 signaling and phosphorylation of SYK in macrophage-like differentiated human THP-1 cells (Fig. 6E-F). TREM2 ko cells, used as a negative control, did not respond to the incubation with either anti-TREM2 antibody. In contrast, THP-1 wt cells showed a 2-fold increase in the levels of phosphorylated SYK (pSYK) upon stimulation with monoclonal antibody 4B2A3 and a more than 4-fold increase with polyclonal antibody AF1828. Interestingly, consistent with the significant decrease of TREM2 and DAP12 expression levels, neither antibody stimulated the phosphorylation of SYK in RER1 ko cells (Fig. 6E-F). Very similar results were obtained when phosphorylated SYK and total SYK levels were detected by western immunoblotting (Fig. 6F), rather than AlphaLISA (Fig. 6E), upon cell treatment with antibodies 4B2A3 or AF8128. Together, these combined data further support a loss-of-function in the TREM2-DAP12 signaling capacity in RER1 ko cells.

Next, it was assessed whether RER1 deletion also affects phagocytosis of pHrodo-labeled *E.coli* bioparticles (Fig. 6G). Fluorescence readings over a period of 5 h indicated efficient phagocytosis of these particles by macrophage-like differentiated THP-1 wt cells. As expected, phagocytosis was strongly reduced in TREM2 ko cells. More importantly, the deletion of RER1 also resulted in almost complete inhibition of phagocytic activity (Fig. 6G-H).

## Discussion

The presented data indicate that RER1 prevents cell surface transport of DAP12 when it is not assembled with one of its cognate immunoreceptors, and that unassembled DAP12 is directed to proteasomal degradation. Thus, RER1 is critically involved in the regulation of DAP12 mediated signaling and immune cell function.

DAP12 plays important roles in the regulation of immune cell functions, including survival and proliferation, phagocytosis, chemotaxis, and cytoskeletal remodeling and cytokine production [19–22]. The physiological importance of DAP12 is demonstrated by loss-of-function mutations that cause NHD, a severe disorder characterized by bone abnormalities and progressive dementia [27]. Notably, NHD can also be caused by mutations in TREM2, indicating that impaired TREM2-DAP12 signaling underlies the development of this disease. The interaction of DAP12 and TREM2 is mediated via charged amino acid residues in their TMDs. Previous flow cytometry studies with Ba/F3 cells also indicated that overexpressed DAP12 is not efficiently transported to the cell surface without overexpression of CD94/NKG2C, suggesting a retention mechanism for DAP12 when it is not properly complexed with an immunoreceptor [3]. It was also suggested that DAP12 could facilitate surface transport of its co-immunoreceptor, including TREM2 and KIR2DS [11, 29]. However, overexpressed TREM2 is also transported to the cell surface and secreted in the absence of DAP12 [30, 31]. Thus, DAP12 might not be generally necessary for the surface transport of interacting receptors.

Here, we also explored the effects of TREM2 on the metabolism of DAP12 using a THP-1 differentiated macrophage-like cell model with endogenous expression of TREM2 and DAP12. Interestingly, deletion of TREM2 strongly increased the degradation of DAP12 dimers, indicating destabilization of DAP12 when it is not assembled with an appropriate immunoreceptor. Similar results were also obtained in a non-immune related HEK293 Flp-In cell model with stably overexpressed DAP12 and/or TREM2, indicating that the stabilization of DAP12 by TREM2 does not require other factors expressed in immune cells. Our data with specific inhibitors indicate that the degradation of unassembled DAP12 involves proteasomal rather than lysosomal activity. The degradation of TREM2 remains to be analyzed in detail. The levels of endogenous full-length TREM2 decreased upon treatment of THP-1 cells with proteasome inhibitors. Thus, it is unlikely that full-length TREM2 is a substrate for proteasomal degradation. As TREM2 CTFs were rather increased upon proteasome inhibition, it is possible that the proteasome contributes to their degradation. However, TREM2 CTFs are generated by ectodomain shedding, and can be processed further by γ-secretase [45, 56–58], and it remains to be tested whether these pathways are affected by proteasomal inhibition under our experimental conditions.

Interestingly, DAP12 wt formed increased levels of SDS resistant trimers and tetramers when expressed in the absence of TREM2 in transiently transfected HEK293 cells (Fig. 5A). A similar observation was made previously by Call et al. when studying the assembly of DAP12 with the natural killer cell activating receptor KIR2DS2 using *in vitro* translation experiments [28], and it had been proposed that DAP12 interacting immunoreceptors compete with additional DAP12 molecules for binding to covalently linked DAP12 dimers during complex assembly. In the present study, trimers of endogenously expressed DAP12 were detectable in TREM2 ko macrophage-like differentiated THP-1 cells upon inhibition of the proteasome. Proteasomal inhibition also led to the detection of trimeric DAP12 in HEK293 cells stably overexpressing DAP12 with the TREM2 K186N mutant, further supporting the notion that TREM2 or other receptors compete with monomeric DAP12 for binding to covalently linked DAP12 dimers.

We also noticed that DAP12 is not transported to the cell surface in the absence of TREM2 or when the interaction with TREM2 is prevented by mutating lysine residue K186 in the TMD of TREM2 that is critical for the interaction with DAP12. Immunocytochemistry also revealed that unassembled DAP12 is mainly retained in compartments of the early secretory pathway, including the ER and ERGIC. Interestingly, the DAP12 D50A variant was found in post ER compartments and at the cell surface also in the absence of TREM2, indicating that the charged aspartate residue in the TMD is important for the retention of unassembled DAP12 (Fig. 4). Similar observations were made previously using flow cytometry of Ba/F3 cells virally transduced with DAP12 constructs [3]. Indeed, charged or polar amino acid residues in TMDs could be important determinants in the ER retention of several membrane proteins [52].

RER1 is a sorting receptor for the retrieval of ER membrane proteins and selected unassembled subunits of larger protein complexes. It can retrieve clients from early Golgi compartments by recognizing charged or other polar amino acid residues within the respective transmembrane domains. Human client proteins of RER1 include unassembled presenilin enhancer 2 (PEN2), immature nicastrin, the α-units of acetylcholine receptors (AChR) and the peripheral myelin protein 22 (PMP22) [33–35, 59]. Additional RER1 client proteins have been identified, including the ER resident membrane proteins Sec12p, Sec71p, Sec63p [60, 61], and unassembled subunits of oligomeric membrane complexes like Fet3 [62]. By co-immunoprecipitation assays, we could demonstrate that RER1 interacts with DAP12, and that the charged D50 residue in the DAP12 TMD is critical for this interaction (Fig. 5A). Thus, these combined results provide evidence for a molecular mechanism mediating the retention and regulated degradation of unassembled DAP12. We also observed that DAP12 and RER1 interacted independently of the presence of TREM2 in THP-1 cells, whereas in HEK293 cells with the overexpression of DAP12 and TREM2 mutants, this interaction was observed only in the absence of TREM2.

Thus, a pool of unassembled DAP12 might exist in THP-1 cells that is stabilized by the interaction with RER1 in early secretory compartments, and is available on demand for assembly with TREM2 or other immunoreceptors capable to interact with DAP12. In the HEK293 cell model, we used a bicistronic construct encoding TREM2 and DAP12 linked by a T2A sequence to assure stoichiometric expression of both proteins. Thus, in this model likely most of DAP12 (dimers) associate with TREM2 thereby precluding interaction with RER1. However, the relative contribution of RER1 to the retention/retrieval of unassembled DAP12 and the exact molecular mode of interaction of both proteins remains to be dissected in more detail.

A functional relevance of RER1 in the expression and signaling of TREM2-DAP12 complexes was demonstrated by the deletion of the RER1 gene in THP-1 cells. As compared to wt cells, RER1 ko cells showed strongly decreased levels of both TREM2 and DAP12 (Fig. 6). It has been reported previously that knockdown of RER1 results in decreased levels of the AchR α-subunit, which is also a client protein of RER1 [34]. These findings suggest that RER1 not only controls the retention or retrieval of unassembled components of certain membrane protein complexes, but also may stabilize these proteins until they properly assemble into functional complexes prior to transport to the cell surface to interact with specific ligands and trigger membrane proximal signaling.

Indeed, RER1 ko THP-1 cells showed strongly reduced phosphorylation of SYK upon stimulation of TREM2 with agonistic antibodies. Furthermore, the deletion of the RER1 gene in THP-1 cells significantly decreased phagocytic activity, thereby showing the importance of RER1 in the maintenance of immune cell function.

RER1 is almost ubiquitously expressed in different cell types [63]. An important function of RER1 is the modulation of Notch signaling during mouse cerebral cortex development by maintaining sufficient cell surface expression and activity of the γ-secretase complex [64]. RER1 has also been recognized as one of the most stably expressed genes throughout choroid plexus development and ageing in mice, and considered as a housekeeping gene [65]. Abundant expression of RER1 mRNA and protein is also detected in microglia and other immune cells [66–68]. γ-Secretase dependent Notch signaling also regulates lymphocyte development, differentiation and proliferation [69], and the inflammatory response of myeloid cells to various stimuli [70, 71]. The present data show that RER1 plays additional roles in the regulation of immune cell function by controlling assembly and surface expression of functional TREM2-DAP12 complexes. Thus, it will be important to further dissect RER1 function in immune cells, and its role in the regulation of inflammatory processes.

## Conclusions

The combined results indicate that unassembled DAP12 is retained in early secretory compartments by a mechanism involving RER1. The retention of DAP12 likely plays a critical role in control of proper assembly of functional immunoreceptor complexes before their transport to the plasma membrane. Studies with RER1 knockout cells indeed revealed a functional role of RER1 in the regulation of TREM2-DAP12 complex levels and their signaling capacity at the cell surface. These identified molecular mechanisms regulating TREM2-DAP12 complex assembly and function at the post-translational level may modulate immune cell function and neuroinflammation.

### Electronic supplementary material

Below is the link to the electronic supplementary material.

Supplementary Material 1

## Data Availability

Original data and materials are available upon reasonable request.

## Abbreviations

AChR: Acetylcholine Receptor
APP: Amyloid-Precursor-Protein
BSA: Bovine Serum Albumin
CHX: Cycloheximide
CrRNA: CRISPR RNA
CTFs: C-Terminal Fragments
Cyto D: Cytochalasin D
DAP12: DNAX-activating protein of 12 kDa
DAPI: 4′,6-Diamidino-2-Phenylindole
DTT: Dithiothreitol
DMEM: Dulbecco’s Modified Eagle Medium
ER: Endoplasmic reticulum
ERGIC: ER-Golgi Intermediate Compartment
FBS: Fetal Bovine Serum
HEK293 cells: Human Embryonic Kidney 293 cells
HPRT1: Hypoxanthine Phosphoribosyltransferase 1
IP: Immunoprecipitation
ITAM: Immunoreceptor Tyrosine based Activating Motif
KIR2DS: The two-domain-stimulatory killer Ig-like receptor
Ko: Knock out
NHD: Nasu-Hakola disease
P/S: Penicillin/Streptomycin
PBS: Phosphate Buffer Saline
PBST: Phosphate-Buffered Saline, 0.1% Tween® 20 Detergent
PFA: Paraformaldehyde
PLL: Poly-L-lysine
PMA: Phorbol 12-Myristate 13-Acetate
PMP22: Peripheral Myelin Protein 22
PEN2: Presenilin Enhancer 2
pSYK: Phosphorylated sleen tyrosine kinase
qRT-PCR: Quantitative Reverse Transcription PCR
RER1: Retention in ER Sorting Receptor 1
RNP: Ribonucleoprotein
RT: Room Temperature
sgRNAs: Single guide RNAs
SYK: Spleen Tyrosine Kinase
TBST: Tris-Buffered Saline, 0.1% Tween® 20 Detergent
tracrRNA: Transactivating crRNA
TREM2: Triggering Receptor Expressed on Myeloid cells 2
TYROBP: Tyrosine kinase-Binding Protein
UBC: Ubiquitin C
wt: Wild type
