# Supplementary material for: DAP12 interacts with RER1 and is retained in the secretory pathway before assembly with TREM2
Source: Cell Mol Life Sci. 2024 Jul 15;81(1):302. doi: 10.1007/s00018-024-05298-w (PMC11335228; doi:10.1007/s00018-024-05298-w)
Supplement: Supplementary file 1 — Supplementary Material 1 [file 18_2024_5298_MOESM1_ESM.docx]

**Supplementary information**

**Supplementary Figure 1**


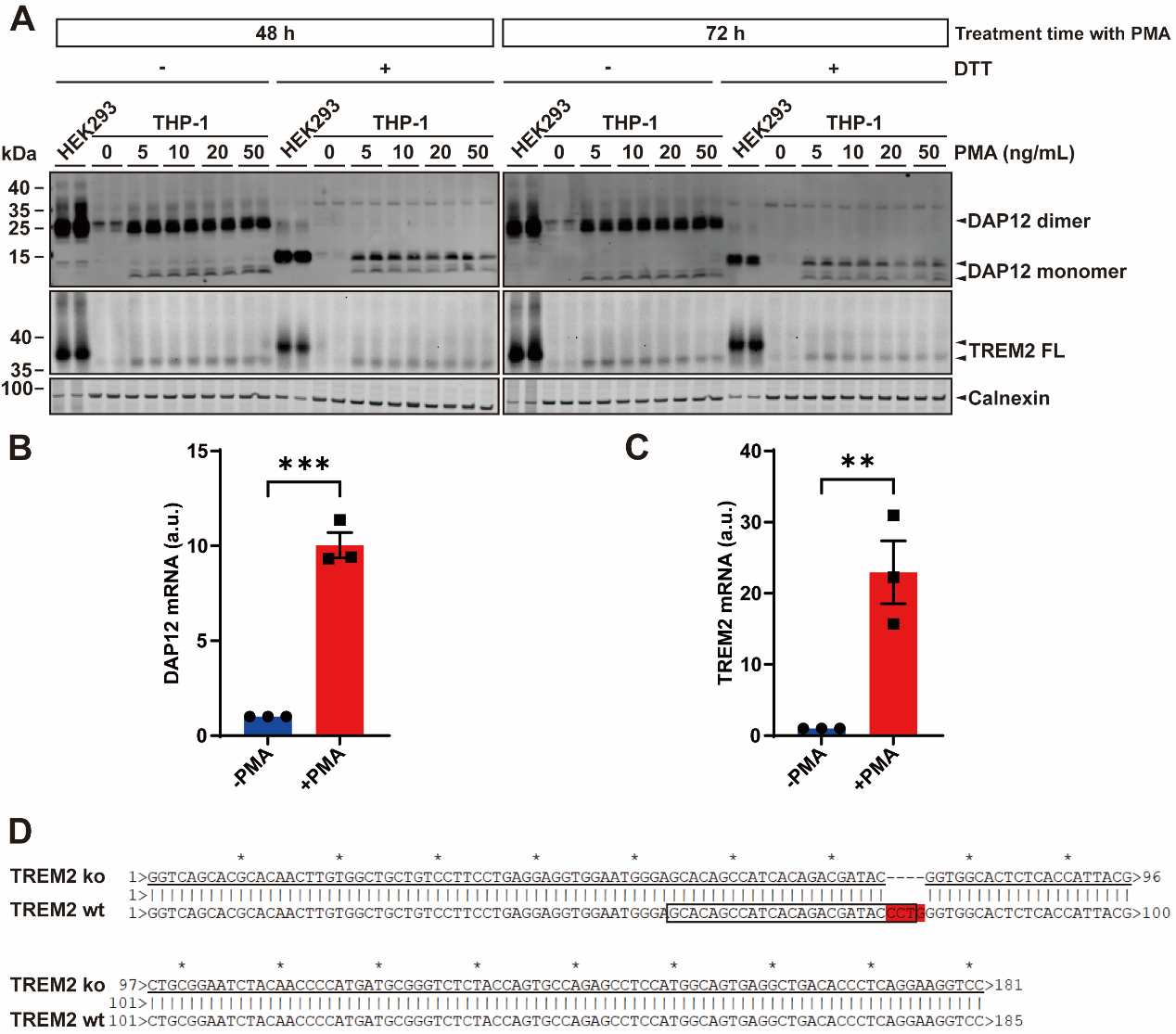


**Suppl. Fig. 1: Differentiation of THP-1 cells into macrophage-like cells increases endogenous expression of TREM2 and DAP12**

(A) Comparison of the DAP12 and TREM2 expression levels in THP-1 wt cells differentiated with PMA at the indicated concentrations and incubation times followed by one day recovery in normal RPMI 1640 after PMA removal. HEK293 cells transiently transfected with the bicistronic TREM2 wt+DAP12 wt construct served as positive control. Membrane proteins were isolated, and samples prepared under reducing (with dithiothreitol (DTT)) or non-reducing (without DTT) conditions, and analyzed by western immunoblotting. TREM2 FL: TREM2 full-length protein.

(B) and (C) Comparison of the DAP12 and TREM2 mRNA levels in differentiated (5 ng/mL PMA, 48 h + 1 day recovery) and undifferentiated THP-1 wt cells by real-time qRT-PCR. Values represent Mean ± SEM of three independent experiments. Each data point represents the mean value of an individual experiment. Student’s t-test (unpaired, two-tailed). ***p* <0.01, ****p* <0.001.

(D) DNA sequence of TREM2 in CRISPR/Cas9-mediated THP-1 TREM2 ko cells. DNA was extracted and the primer 5’-GTGTCTTGCCCCTATGACTCC-3’ used for DNA sequencing. The black open rectangle indicates the sequence of the gRNA used for TREM2 targeting. The sequence highlighted in red indicates the deleted sequence in the TREM2 gene.

**Supplementary Figure 2**


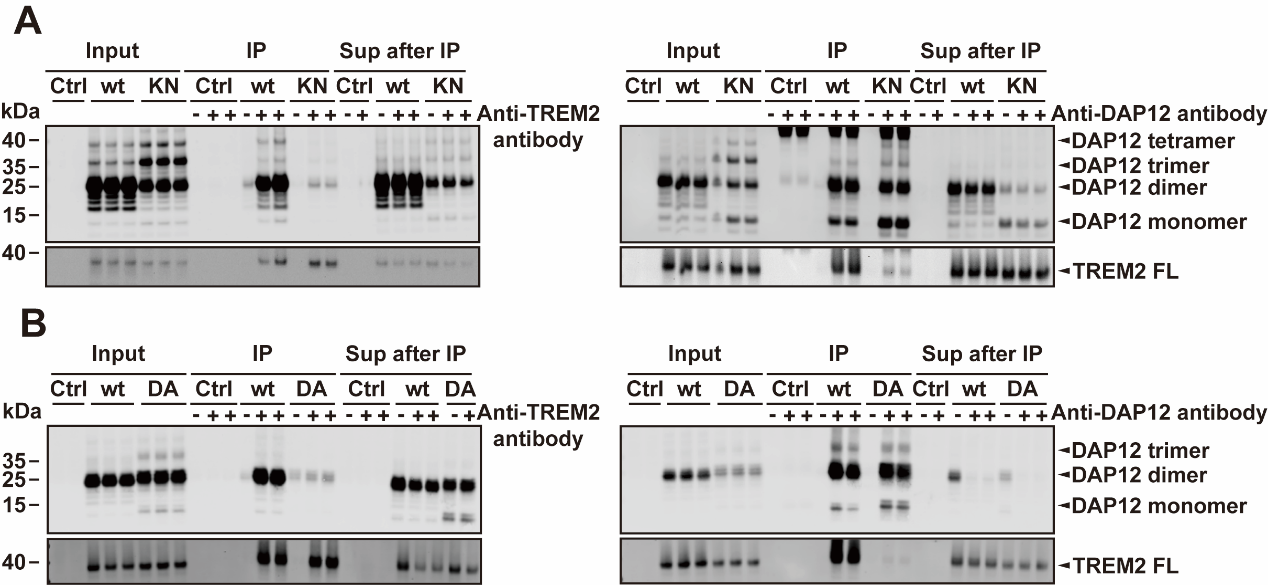


**Suppl. Fig. 2: Analysis of the interaction between different TREM2 and DAP12 variants**.

(A) HEK293 cells were transiently cotransfected with TREM2 wt+DAP12 wt (wt) or TREM2 K186N+DAP12 wt (KN). Non-transfected HEK 293 cells served as control (Ctrl). Membrane proteins were extracted and subjected to co-immunoprecipitation with anti-TREM2 antibody (4B2A3) or anti-DAP12 antibody (DAP12.2). TREM2 FL: TREM2 full-length protein.

(B) HEK293 cells were transiently cotransfected with TREM2 wt+DAP12 wt (wt) and TREM2 wt+DAP12 D50A (DA). Non-transfected HEK 293 cells served as control (Ctrl). Non-transfected HEK 293 cells served as control (Ctrl). Membrane proteins were extracted and subjected to co-immunoprecipitation with anti-TREM2 antibody (4B2A3) or anti-DAP12 antibody (DAP12.2). TREM2 FL: TREM2 full length protein.

**Supplementary Figure 3**


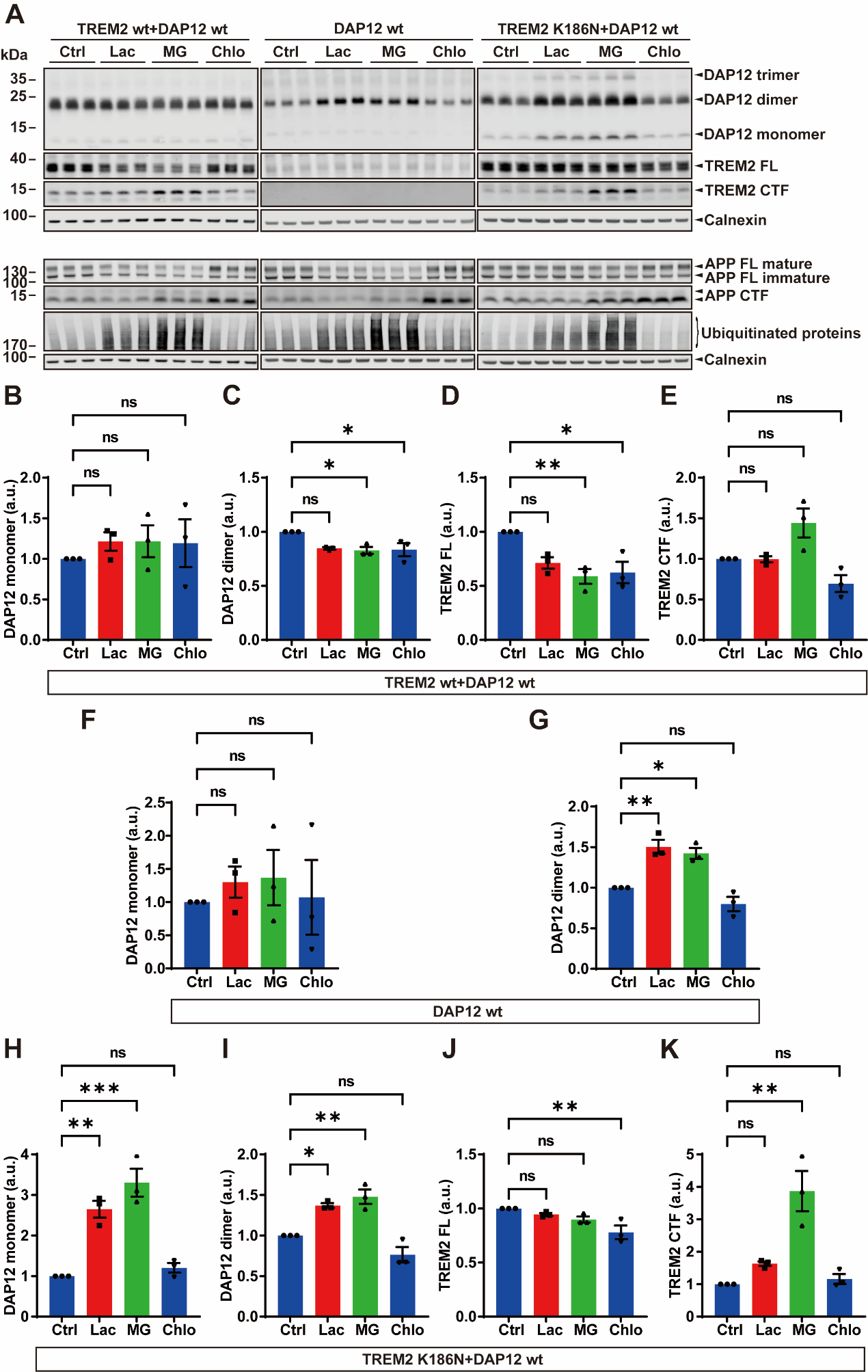


**Suppl. Fig. 3: Characterization of degradation pathways for DAP12 and TREM2 in HEK293 cells overexpressing TREM2 and DAP12 variants.**

(A) Cells overexpressing DAP12 wt alone or in combination with TREM2 wt or the TREM2 K186N mutant were treated with MG132 (MG, 10 μM), lactacystin (Lac, 10 μM), chloroquine (Chlo, 50 μM) for 4 h. Control cells (Ctrl) were left untreated. Cellular membranes were isolated and the indicated proteins detected by western immunoblotting. Ubiquitinated proteins and amyloid precursor protein (APP) were detected as positive controls for efficient inhibition of proteasomal and lysosomal activity, respectively, TREM2 FL: TREM2 full length protein; TREM2 CTF: TREM2 C-terminal fragment; APP FL: APP full-length protein; APP CTF: APP C-terminal fragment.

(B) - (K) Quantification of DAP12 monomer, dimer, TREM2 FL and CTFs levels. (B) DAP12 monomer in TREM2 wt-DAP12 wt expressing cells; (C) DAP12 dimer in TREM2 wt-DAP12 wt expressing cells; (D) TREM2 FL in TREM2 wt-DAP12 wt expressing cells; (E) TREM2 CTF in TREM2 wt-DAP12 wt expressing cells; (F) DAP12 monomer in DAP12 wt expressing cells; (G) DAP12 dimer in DAP12 wt expressing cells; (H) DAP12 monomer in TREM2 K186N-DAP12 wt expressing cells; (I) DAP12 dimer in TREM2 K186N-DAP12 wt expressing cells; (J) TREM2 FL in TREM2 K186N-DAP12 wt expressing cells; (K) TREM2 CTF in TREM2 K186N-DAP12 wt expressing cells, Indicated proteins were normalized to calnexin. Mean ± SEM of three independent experiments each performed with triplicate samples. Each data point represents the mean of one experiment. One way ANOVA (post hoc Tukey's multiple comparisons test). **p* <0.05, ***p* <0.01, ****p* <0.001. Lactacystin and MG132 slightly decreased the levels of full-length TREM2 in HEK293 cells co-expressing DAP12 with TREM2 wt or TREM2 K186N. MG132 significantly increased levels of the TREM2 CTF of cells expressing the K186N mutant. Chloroquine significantly decreased the levels of TREM2 FL but had no effect on the levels of TREM2 CTFs in both cells co-expressing DAP12 together with TREM2 wt or with the TREM2 K186N mutant.

**Supplementary Figure 4**


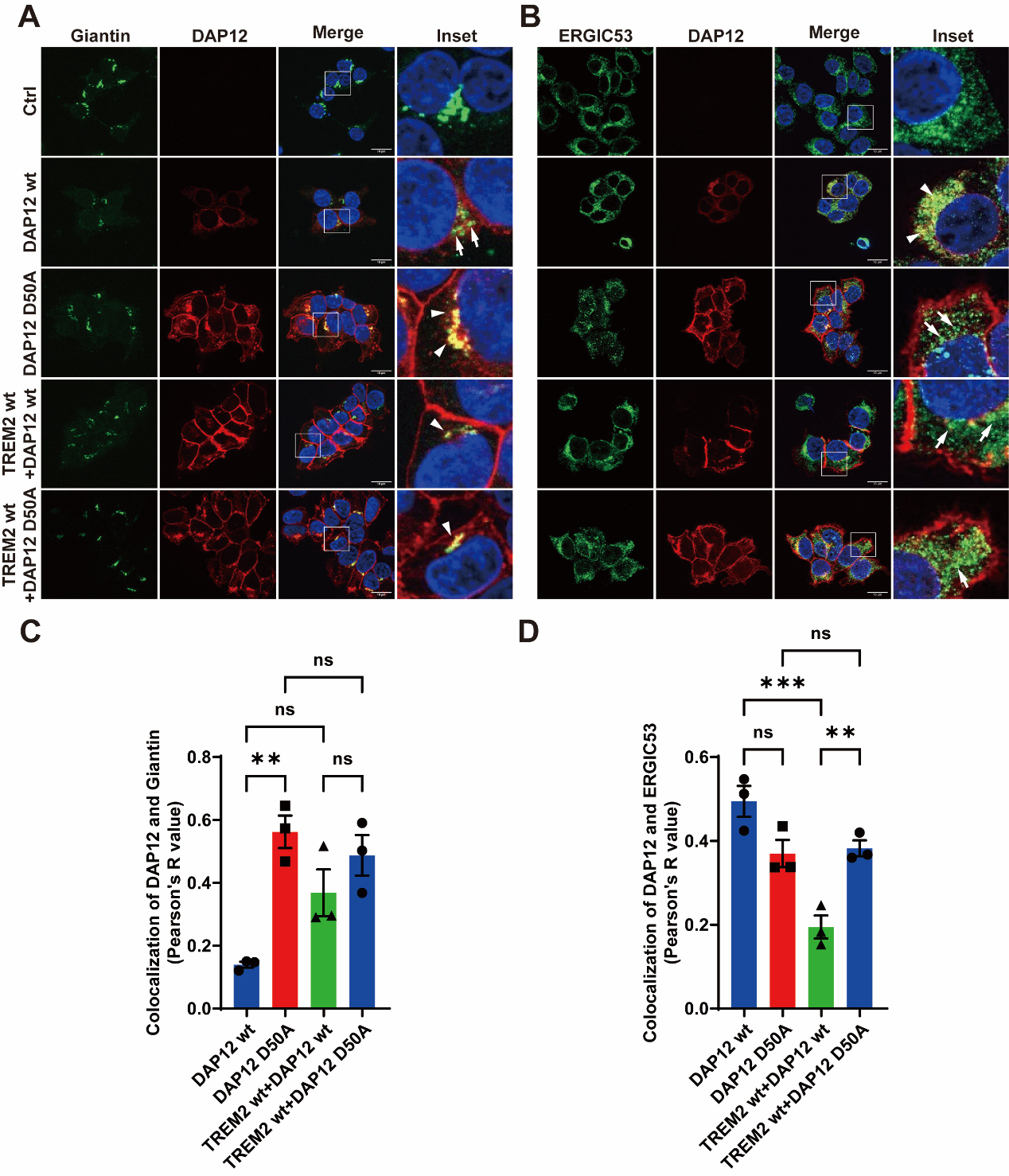


**Suppl. Fig. 4: Subcellular localization of DAP12 in HEK293 cells stably expressing DAP12 variants with or without TREM2.**

(A) - (B) Immunocytochemical detection of DAP12 in HEK293 cells stably expressing DAP12 wt or DAP12 D50A alone or together with TREM2 wt. Non-transfected HEK293 Flp-In cells (Ctrl) served as control. Shown are representative images. DAP12 (D7G1X antibody) is shown in red (A, B), Giantin (A) and ERGIC53 (B) are shown in green. Nuclei were counterstained with DAPI. Arrowheads indicate colocalization of DAP12 with the respective marker protein. Arrows indicate separate localization of DAP12 from the respective marker protein. Scale bar =10 μm.

(C) - (D) Pearson’s R value of colocalization of DAP12 with the indicated maker proteins for the cis- and medial-Golgi network (Giantin) or the ER Golgi intermediate compartment (ERGIC). Values represent Mean ± SEM of three independent experiments. Each data point represents the mean value of an individual experiment. One-way ANOVA (post hoc Tukey's multiple comparisons test). ***p* <0.01, ****p* <0.001.

**Supplementary Figure 5**


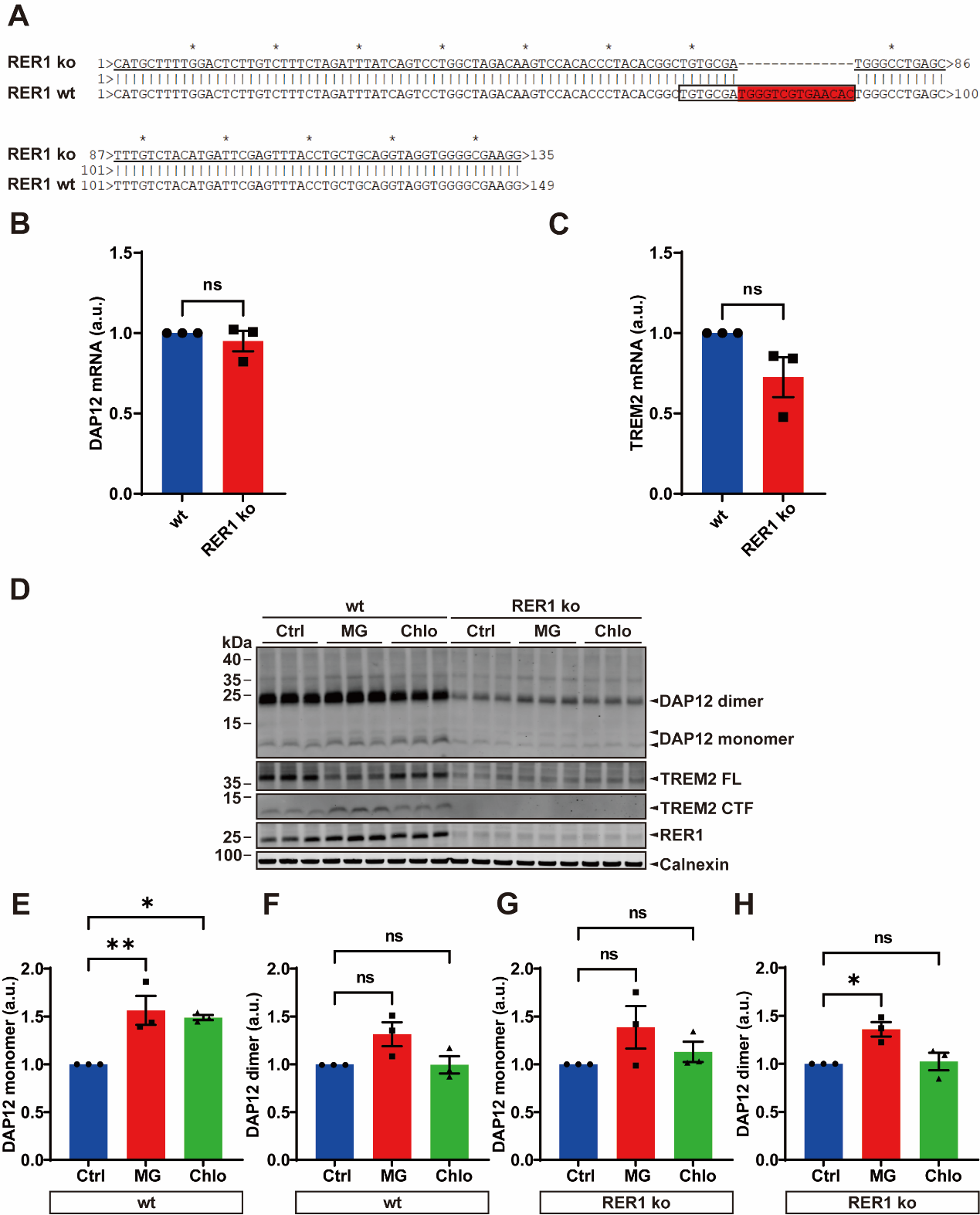


**Suppl. Fig. 5: Increased degradation of DAP12 in RER1 knockout THP-1 cells**

(A) DNA sequence of RER1 in CRISPR/Cas9-mediated THP-1 RER1 ko cells. DNA was extracted and the primer 5’-ACATCACGCCCAGGTAACG-3’ used for DNA sequencing. The black open rectangle indicates the sequence of the gRNA used for RER1 targeting. The sequence highlighted in red indicates the deleted sequence in the RER1 gene.

(B) - (C) Quantification of DAP12 and TREM2 mRNA levels in differentiated THP-1 wt and RER1 ko cells by real-time qRT-PCR. Values represent Mean ± SEM of three independent experiments. Each data point represents the mean value of an individual experiment. Student’s t-test (unpaired, two-tailed).

(D) Characterization of degradation pathways for DAP12 and TREM2. THP-1 wt and RER1 ko cells were treated with MG132 (MG, 10 μM) or chloroquine (Chlo, 50 μM) for 4 h. Untreated cells served as control (Ctrl). Proteins were extracted from isolated membranes and analyzed by western immunoblotting. TREM2 FL: TREM2 full length protein; TREM2 CTF: TREM2 C-terminal fragment.

(E) - (H) Quantification of DAP12 levels by western immunoblotting in (D). Dimeric and monomeric DAP12 were normalized against calnexin. Values represent Mean ± SEM of three independent experiments. Each data point represents the mean value of an individual experiment. One way ANOVA (post hoc Tukey's multiple comparisons test). **p* <0.05, ***p* <0.01.
